# Probing Natural Killer Cell Education by Ly49 Receptor Expression Analysis and Computational Modelling in Single MHC Class I Mice

**DOI:** 10.1371/journal.pone.0006046

**Published:** 2009-06-25

**Authors:** Sofia Johansson, Mali Salmon-Divon, Maria H. Johansson, Yishai Pickman, Petter Brodin, Klas Kärre, Ramit Mehr, Petter Höglund

**Affiliations:** 1 Department of Microbiology Tumor and Cell Biology, Karolinska Institute, Stockholm, Sweden; 2 EMBL, European Bioinformatics Institute, Wellcome Trust Genome Campus, Cambridge, United Kingdom; 3 The Mina and Everard Goodman Faculty of Life Sciences, Bar-Ilan University, Ramat-Gan, Israel; Centre de Recherche Public de la Santé (CRP-Santé), Luxembourg

## Abstract

Murine natural killer (NK) cells express inhibitory Ly49 receptors for MHC class I molecules, which allows for “missing self” recognition of cells that downregulate MHC class I expression. During murine NK cell development, host MHC class I molecules impose an “educating impact” on the NK cell pool. As a result, mice with different MHC class I expression display different frequency distributions of Ly49 receptor combinations on NK cells. Two models have been put forward to explain this impact. The two-step selection model proposes a stochastic Ly49 receptor expression followed by selection for NK cells expressing appropriate receptor combinations. The sequential model, on the other hand, proposes that each NK cell sequentially expresses Ly49 receptors until an interaction of sufficient magnitude with self-class I MHC is reached for the NK cell to mature. With the aim to clarify which one of these models is most likely to reflect the actual biological process, we simulated the two educational schemes by mathematical modelling, and fitted the results to Ly49 expression patterns, which were analyzed in mice expressing single MHC class I molecules. Our results favour the two-step selection model over the sequential model. Furthermore, the MHC class I environment favoured maturation of NK cells expressing one or a few self receptors, suggesting a possible step of positive selection in NK cell education. Based on the predicted Ly49 binding preferences revealed by the model, we also propose, that Ly49 receptors are more promiscuous than previously thought in their interactions with MHC class I molecules, which was supported by functional studies of NK cell subsets expressing individual Ly49 receptors.

## Introduction

NK cells efficiently lyse target cells lacking expression of MHC class I molecules, but usually spare cells expressing sufficient levels of self-MHC. The inhibitory impact by MHC class I is conveyed by inhibitory receptors, of which KIR receptors in humans and Ly49 receptors in mice are the most important [1]–[3]. CD94/NKG2 heterodimers, specific for nonclassical MHC class Ib molecules loaded with peptides derived from some MHC class Ia alleles, exist in both species and provide an indirect way for NK cells to detect loss of classical self MHC class I [4]. The balance between activating and inhibitory receptors determines the outcome of the NK cell-target cell encounter.

Ly49 receptors may share specificities for some MHC class I alleles but discriminate sharply between others. As Ly49 and MHC class I genes are located on different chromosomes [5], and allelic polymorphisms in the MHC class I locus largely exceeds that of the Ly49 gene cluster, it has been suggested that NK cells must adapt to the MHC environment in order to ensure missing self specificity for host MHC class I [6]–[8].This adaptaion is mostly acting at the level of co-expression of several Ly49 receptors on the same NK cell [9]–[18]. The mechanisms by which self MHC modulates the NK cell receptor repertoire are not known, but could include a direct impact on Ly49 expression on individual NK cells, or by a selection mechanism favoring the survival (or proliferation) of NK cells with appropriate Ly49 receptors.

Studies of MHC class I deficient [19], [20] and mosaic [21]–[23] mice shown that NK cell tolerance is secured even in the absence of appropriate Ly49/MHC class I interactions. In such situations, NK cells could survive as hyporesponsive or anergic cells [24]–[26]. Thus, there are at least two processes, with different measurable endpoints, that operate at the cellular level to ascertain NK cell tolerance. The first would then affect the expression frequencies of cells with different Ly49 receptors, i.e. the “Ly49 repertoire”, while the second would rather adapt the activation status of each NK cell without altering the frequencies of cells with defined Ly49 receptor combinations. In this study, we address the first process.

Raulet and colleagues have suggested two developmental schemes to describe how the Ly49 repertoire may be determined during NK cell education [27]. In both schemes, Ly49 genes are assumed to be stably activated, i.e. once they have been activated they stay on. In the first model, the sequential model, developing NK cells express new Ly49 genes continuously and cumulatively, but in a random order. During development, each NK cell will be periodically tested for interactions with self MHC class I molecules on neighbouring cells and the cell will mature as soon as it expresses inhibitory receptors with sufficient cumulative interaction strengths to self MHC class I molecules. The alternative model is a two-step selection model that proposes that the Ly49 repertoire is fully formed already at the initial stage, by a stochastic process, and subsequently shaped by two selection steps: one selecting for cells expressing self-specific Ly49 receptors, and the other selecting against cells expressing too many self-specific receptors.

Mathematical modelling has the power to generate an infinite number of Ly49 repertoires, using different models and/or parameter values. These can then be fit to experimental data. Such an approach can be used to differentiate between the two models. In previous studies [28], [29], we used computer simulations to show that each of these two education mechanisms gave a better fit to Ly49 expression patterns than the “product rule”, which assumes no influence of MHC on Ly49 receptor frequencies [27]. However, the biological data which those studies were based on only included measurements of Ly49 receptors in pairs, which was insufficient to decidedly favour one model over the other. Here, we present a new analysis on Ly49 receptor expression frequencies, measured in triplets, in four mouse strains expressing different single MHC class I alleles. These new data was used in combination with mathematical modelling, allowing us to draw novel conclusions as to the mechanisms of NK cell education.

## Results

### Computer simulations of NK cell receptor repertoires in single-MHC class I mice

In our previous work, we created two computer models that simulated potential education steps according to either the sequential hypothesis or the two-step selection hypothesis [28], [29]. The two models were here independently modified to simulate NK cell development on four single MHC class I backgrounds: H2K^b^, H2D^b^, H2D^d^ and H2L^d^ mice [18]. We focused on four Ly49 receptors (Ly49A, -C, G2, and -I), which were modeled three at a time, to simulate the experiments described below.

One prerequisite for these simulations was that the expression frequencies of receptors in the absence of education (the pre-education repertoire) must be known. We measured these frequencies in MHC^−^ mice, since no MHC-based education can take place on this background [27]. Several parameters that are important for this educational process are, however, unknown. Thus they needed to be changed between runs of the simulations and tested individually. Table 1 lists the parameters used in our simulations. Of those, we would particularly like to emphasize that the binding strength between different MHC class I molecules and Ly49 receptors was varied as a parameter and not assumed to take any given value based on previous data. We have thus not assigned a priori which Ly49 receptors binds to “self” MHC class I and which do not, but let the simulations test these interactions impartially. Apart from the three receptors that were taken into account in each simulation run, additional receptors may affect education. To encompass this possibility, a 4^th^ general receptor was added in each simulation. This receptor can in fact represent all educating effects that are not considered in the specific modelling scheme.

**Table 1:** Simulation parameters, constant values and ranges used.

Parameter name	Definition	Constant value	Range in simulations
Receptor binding strength	Binding strength between each Ly49 receptor (including the 4^th^ receptor) and each MHC class I allele	–	0–4 (in intervals of 1)
Ly49 pre-selection probabilities*	Ly49A	0.19	–
	Ly49C	0.30	–
	Ly49G2	0.55	–
	Ly49I	0.64	–
	4^th^ receptor	–	0–1 (in intervals of 0.1)
S*_min_*	Minimum maturation threshold	–	0–16 (in intervals of 1)
S*_max_* **	Maximum maturation threshold	–	0–16 (in intervals of 1)

* The constant values were calculated from the expression frequencies of each receptor in MHC deficient mice. The expression probability of the 4^th^ receptor was varied as a parameter.

** This parameter was used only in the two step selection model.

**Figure 1:**
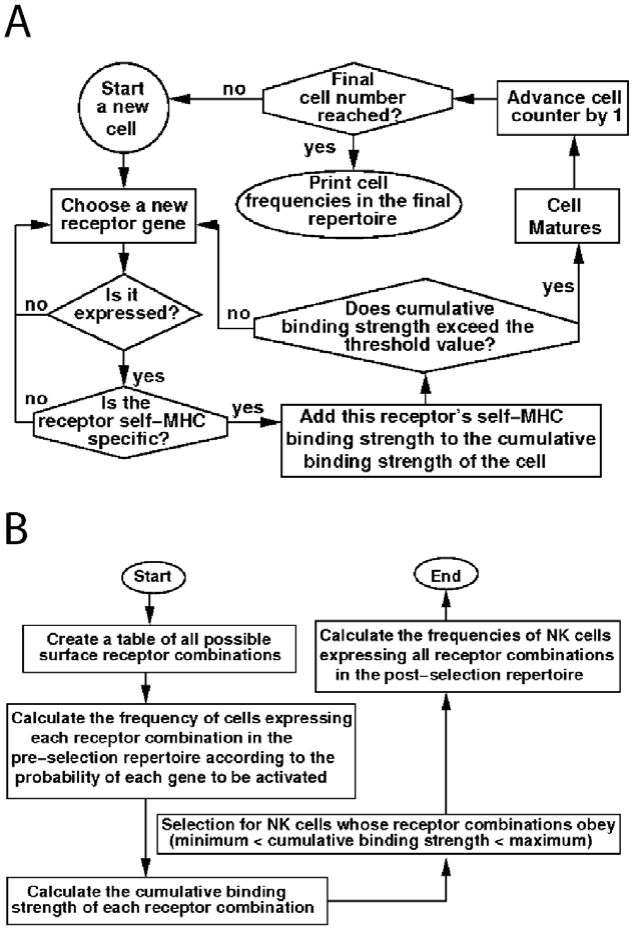
Description of the models. Flow chart of the sequential model simulation (A), and the two-step selection program (B). In each run, the parameters of the simulation are fixed throughout the run. The simulations were each run for a large number of parameter sets (1,700,000 sets for the two-step selection simulation and 110,000 sets for the sequential model simulation), for each MHC background and staining. See text for details.

In the sequential simulation (Fig. 1A), each run creates the repertoire cell by cell. The decision to express a receptor or not is taken one by one for each receptor, in a random order, and is based on the expression probability for that receptor (see above). We defined the parameter *S_min_* (Table 1) as the minimal total interaction strength that an NK cell must possess in order to be allowed to mature and become part of the post-education repertoire. If the first tested receptor did not give sufficient interaction strength to pass this threshold, a new receptor was expressed and its interaction strength was added to that of the first receptor, followed by a new comparison to *S_min_*. This cycle was repeated until the cell had either received a survival signal and joined the post-education pool, or exhausted all possible receptors and died. The model then initiates a new NK cell, defines the random order to test the different receptors, and goes through all the steps again. Each simulation run generates 100,000 NK cells, and, in the end of each run, the program calculates the frequencies of cells expressing each receptor pattern in the accumulated post-education repertoire. The computer then changes one of the parameter values by one step and goes through the process again. Each series of runs in this model tested about 110,000 parameter value sets. These were made up from combinations of 17 values of *S_min_*, 4^4^ combinations of interaction strength values and 11 values for the unknown receptor's pre-education expression frequency (Table 1). One such series was performed for each MHC background and each combination of Ly49 receptors that were measured, in total 16 different setups.

In the two-step selection simulation (Fig. 1B), there was no need to simulate individual cells, as the selection is assumed to be separate from the process of receptor expression. Hence, in each run of this simulation, a table of all possible surface expression patterns of the included Ly49 receptors was created. Next, based on the expression probabilities of each Ly49 receptor (taken from MHC^−^ mice as in the sequential model simulation), the frequency of each receptor combination in the pre-education repertoire was determined. Subsequently, the total interaction strength, *S*, of each expression pattern with the self-MHC class I molecule under investigation was calculated. The observed value was then compared to both a lower and an upper interaction strength threshold, *S_min_* and *S_ma_*_x_, where *S_min_* is the minimum total inhibitory receptor interaction strength required for the NK cell to survive and *S_max_* is the maximum total inhibitory receptor interaction strength that still allows the NK cell to survive. *S_max_* was included in this model to allow for the possibility that NK cells with too high affinity for self MHC class I would be selected against [10]. All NK cells whose *S* value was between *S_min_* and *S_max_* would survive. In the end, the program calculated the frequencies of cells expressing each receptor pattern in the post-education repertoire. The relevant parameters in the two-step selection simulations are thus the same as in the sequential model simulation, with the addition of *S_max_*. Each series of runs of the two-step selection model thus tested 1,700,000 parameter sets (the same parameter sets as in the sequential simulations * 17 values of *S_ma_*_x_). One such series of runs was performed for each MHC background and combination of Ly49 receptors.

### Fit to biological data favours the two-step selection model

**Figure 2:**
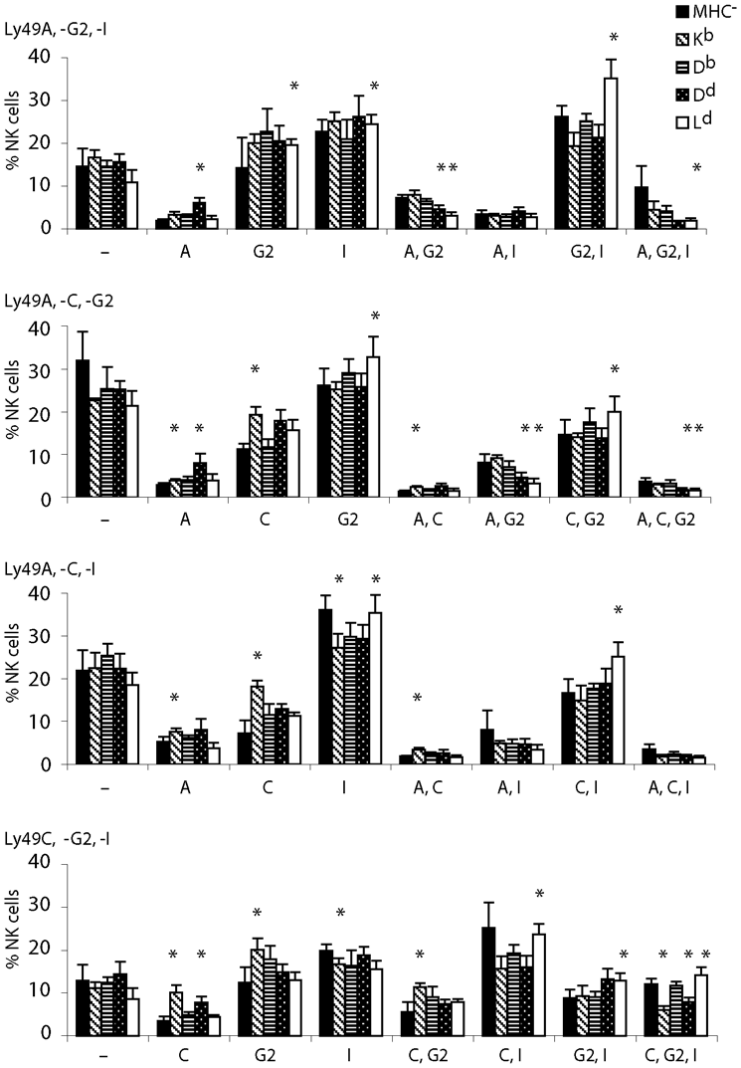
Cell surface expression frequencies (in percentages) of receptor pairs and triplets on NK cells (NK1.1^+^CD3^−^) in single-MHC mice. The expression frequencies shown are averages of measurements in three to six animals. Each panel shows results from a staining by a different trio of antibodies; from top to bottom: (A/G2/I), (A/C/G2), (A/C/I), (C/G2/I). The frequencies are exclusive, that is, e.g., the expression frequency of “A” in the (A/G2/I) staining (top panel) includes only cells that were found to be Ly49A^+^G2^−^I^−^ in that experiment, etc.. Error bars denote the data standard deviations, and asterisks denote that the difference from the frequency of the same receptor combination in MHC^−^ mice is statistically significant (using Student's T test).

The outcome of each simulation run was compared to a biological data set to test which one of the two models would be more likely to reflect NK cell education *in vivo*. To generate these biological data, we determined Ly49 receptor expression frequencies using five color FACS analysis on NK cells from the four single MHC class I mice. In the analysis, we evaluated how many cells expressed none of the three receptors, one receptor but not the others, two receptors but not the third, and finally all three receptors (Fig. 2). This analysis confirmed previous results that NK cells co-expressing Ly49A and Ly49G2 were specifically reduced in H2D^d^ and H2L^d^ mice but not in H2K^b^ and H2D^b^ mice [18]. In addition, we found an overrepresentation of NK cells expressing only one self-specific receptor, i.e. Ly49A single-positive NK cells in H2D^d^ mice (Fig. 2, upper two panels) and Ly49C single-positive NK cells in H2K^b^ mice (Fig. 2, lower three panels).

The output from each simulation run was then compared to the biological data and given a value for the fit, called RMS (see material and methods). The smaller the RMS is, the better the fit. The RMS for the two-step model varied between 1.34 and 3.12, while for the sequential model, the best fit values varied between 3.7 and 6.67 (Table 2). Thus, out of all the 16 cases, the least fitting simulation of the two-step selection model was still better than the best simulation of the sequential model, and this was true for each MHC background and receptor triplet separately. In fact, out of all 16 experiments, the two-step model fit the data significantly better than the sequential model in 14 cases (Table 2, F-test with the FDR correction for multiple comparisons). The major conclusion was thus that the simulations presented here clearly favour the two-step selection model above the sequential model for all 16 simulation series.

**Table 2:** Fit of the two-step model and the sequential model to the experimental data.

Ab combinations				Mouse strain											
				Kb			Db			Dd			Ld		
A	C	G2	I	2-step	Seq	p	2-step	Seq	p	2-step	Seq	p	2-step	Seq	p
•		•	•	2.0340	6.1000	0.0004	2.8200	6.1800	0.0162	2.6100	6.4000	0.0074	2.3200	5.1000	0.0158
•	•	•		1.3400	3.7000	0.0010	2.4820	5.1900	0.0118	2.3200	4.4200	0.0388	2.8000	6.0500	0.0178
•	•		•	2.5600	4.6400	0.0220	2.5100	5.1400	0.0139	3.1200	5.1700	0.0827	2.3300	6.6700	0.0022
	•	•	•	2.7500	5.0700	0.0468	2.5300	5.6400	0.0070	2.5200	5.3500	0.0199	2.0500	4.7700	0.0108

For each MHC class I background and receptor staining, the table gives the RMS value of the best-fit parameter set obtained in simulations of the two-step (2-step) model and the sequential (seq) model, fitted to the experimental data. For each of the 16 experiments, we also give the p-value (p) for testing the significance of difference in the RMS values. The values underlined (14 out of 16 cases) were found to be significant under the FDR correction for multiple comparisons.

### Predicted binding properties of Ly49 receptors from the two models suggest that Ly49 receptors are promiscuous in their interaction with MHC class I molecules

We next scrutinized the parameter value sets in all the best-fit cases of the two models, focussing on the binding strength of each Ly49-MHC class I interaction as well as the threshold to pass selection, S*_min_*. We first determined if binding had occurred (affinity>0) or if the receptor in question was a non-self receptor (affinity = 0). If binding had occurred (affinity>0), we evaluated if it was of sufficient strength to alone allow maturation of the NK cell (affinity≥S_min_) or if it was insufficient in itself to pass selection (>0 but <S_min_), requiring additional receptors to reach *S_min_*. Tables S1 and S2 list all best-fit cases for the two-step and sequential models respectively, along with a summarized classification of individual Ly49-MHC class I interactions as displaying either no binding (“no”), insufficient binding to pass selection (“insuff”) or sufficient binding to pass selection in itself (“suff”). Tables 3 and 4 summarize these results to make comparisons between the models easier. When best-fit cases of the same simulation showed variations in Ly49-MHC class I affinity, both outcomes are indicated (Tables 3 and 4).

**Table 3:** Binding of individual Ly49 receptors to single MHC class I molecules in the two step selection simulations.

Ly49 receptor	Ab combinations				Mouse strain											
					Kb			Db			Dd			Ld		
	A	C	G2	I	no	insuff	suff	no	insuff	suff	no	insuff	suff	no	insuff	suff
A	•	•	•			*		*	*			*				*
	•	•		•			*	*	*				*			*
	•		•	•		*		*	*				*			*
C	•	•	•				*		*				*			*
	•	•		•			*	*	*				*			*
		•	•	•			*	*	*			*			*	
G2	•	•	•			*			*				*	*	*	
	•		•	•		*			*			*				*
		•	•	•			*		*			*			*	
I	•	•		•			*			*		*		*	*	
	•		•	•		*				*		*			*	
		•	•	•			*			*		*			*	

For each receptor triplet, the binding properties between the Ly49 receptors and the individual MHC class I allele used in each independent simulation was retrieved from the cases with the lowest RMS value (table 1). The interactions were analysed and classified as being either nonexistent (none), present but insufficient (insuff) or sufficient (suff) in itself to pass selection. Data from from all individual cases with the lowest RMS values are summarized by stars in this table and are shown independently in table S1. When individual cases in each simulation differed in their outcome, two stars have been entered.

**Table 4:** Binding of individual Ly49 receptors to single MHC class I molecules in the sequential selection simulations.

Ly49 receptor	Ab combinations				Mouse strain											
					Kb			Db			Dd			Ld		
	A	C	G2	I	no	insuff	suff	no	insuff	suff	no	insuff	suff	no	insuff	suff
A	•	•	•		*	*		*	*		*	*		*	*	
	•	•		•	*	*			*				*	*	*	
	•		•	•		*		*	*			*			*	
C	•	•	•				*	*	*				*	*	*	
	•	•		•			*		*			*		*	*	
		•	•	•		*			*			*		*	*	
G2	•	•	•		*	*				*	*	*				*
	•		•	•		*				*		*			*	
		•	•	•		*			*			*			*	
I	•	•		•	*	*			*			*				*
	•		•	•			*	*	*			*			*	
		•	•	•		*			*			*			*	

For each receptor triplet, the binding properties between the Ly49 receptors and the individual MHC class I allele used in each independent simulation was retrieved from the cases with the lowest RMS value (Table 1) in that same way as for the two step selection model (Table 3). Data from from all individual cases with the lowest RMS values are shown independently in table S2.

A first major conclusion from this comparison was that both models predicted the involvement of many more Ly49-MHC class I bindings than expected from previous *in vitro* studies. In fact, there was not a single simulation in which a given Ly49 receptor could be classified exclusively as a “no” in interaction with any one of the four MHC class I alleles included in this study (Tables 3, 4, S1, S2). This result is consistent with the idea that the Ly49 specificity is broader and more promiscuous than previously appreciated. The second major conclusion was that the sequential model predicted weaker and more variable Ly49-MHC class I bindings compared to the two-step selection model, which was reflected by the fact that the number of predictions of sufficient strength to pass selection (“suff”) was 21 for the two-step selection model and only 9 for the sequential model.

### Functional outcome of NK cell education of single Ly49 subsets in single MHC class I mice

**Figure 3:**
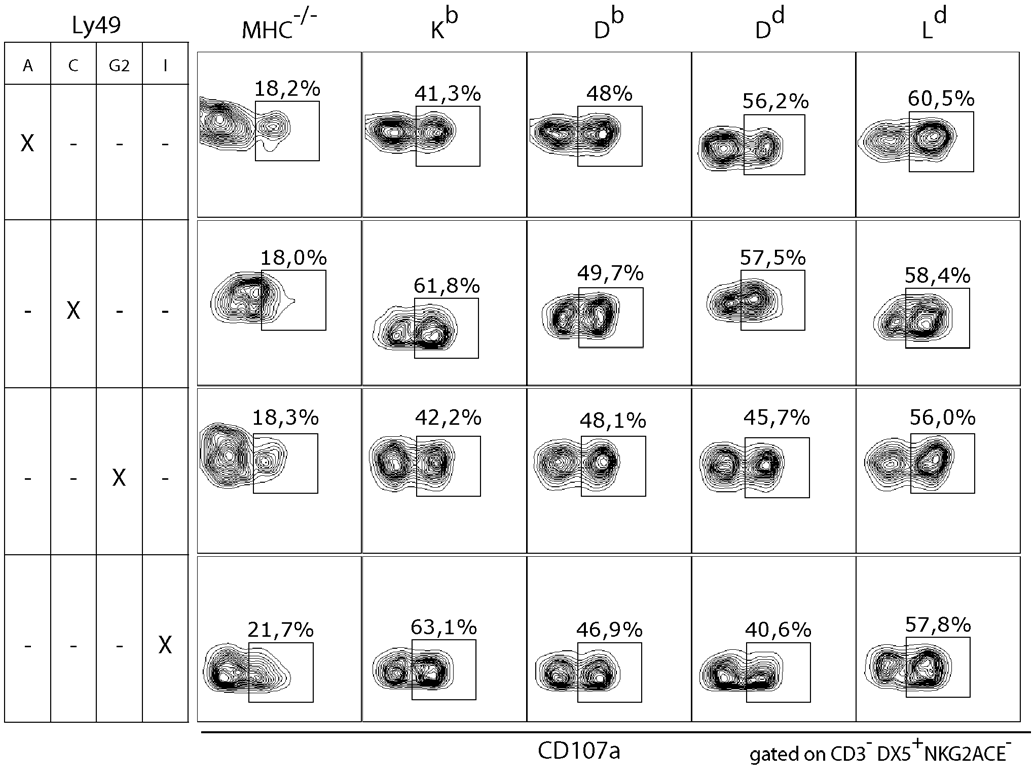
Functional capacity of NK cells from single MHC class I mice expressing only one inhibitory receptor suggest functional outcome of previously unrecognized Ly49-MHC class I interactions. The figures in each plot show percent CD107a^+^ NK cells, expressing only the indicated inhibitory receptor, after stimulation with 50 µg/ml PK136, recognizing the NK1.1 antigen, *in vitro*. One representative experiment out of 3.

Affinity measurements using objective biochemical assays, such as Biacore analysis, does not exist for more than a few Ly49-MHC class I ligand pairs. It is therefore not possible to use the comparison between Tables 3 and 4 to distinguish any further between the two models. However, the surprising observation that all tested Ly49 receptors appeared to bind to all four MHC class I in our simulations nevertheless prompted us to test the functional outcome of NK cell education in the four single MHC class I mice. For this, we used an assay in which the responses of NK cells expressing only one Ly49 receptor could be analysed. If this analysis is done in single MHC class I mice, a comprehensive picture of which Ly49/MHC class I interactions that lead to functional responsiveness would be obtained. We thus developed a 9-color flow cytometry protocol, in which we combined NK cell identification markers (positive and negative) with antibodies against 5 inhibitory NK cells receptors and against one effector function marker for degranulation, CD107a [30]. This analysis showed, surprisingly, that all Ly49 receptors analyzed showed some form of functionality in all single MHC class I mice (Fig. 3), suggesting that MHC class I recognition by Ly49 receptors is promiscuous to an extent not previously appreciated, and as predicted by the modeling in this study.

## Discussion

In this study, the impact of individual MHC class I alleles on the Ly49 receptor repertoire was tested and modelled in mice with single MHC class I alleles. By measuring the expression frequencies of four Ly49 receptors, expressed as singletons, pairs and triplets, we show that each single MHC class I ligand tested has a unique impact on the Ly49 receptor co-expression frequencies, relative to the frequencies in MHC^−^ mice. Furthermore, by comparing these data to results from computational modeling, we found that the two-step selection model for NK cell repertoire formation gave a better fit to the biological data than did the sequential model. Finally, functional experiments suggest that Ly49 receptors may have much broader specificities for MHC class I alleles than previously thought, potentially changing the way we view the process of NK cell education.

A first point of discussion relates to the distribution of Ly49 subsets in our receptor expression analysis. It has been shown that NK cells expressing more than one receptor for self MHC class I are less frequent *in vivo* than would be predicted from the product rule [10], [15]. A presumed reason for this under-representation would be that NK cells with too high affinity for self MHC class I are excluded from the repertoire, as they would be less sensitive to normal cells with downregulated MHC class I expression (missing self recognition). In our FACS analysis, we confirmed that Ly49A^+^G2^+^ NK cells were less frequent in H2D^d^-expressing mice. An interesting novel finding was, however, that NK cells expressing Ly49A or Ly49C as the only receptor (within the triplets investigated here) were more frequent in mice expressing the major ligand, i.e. H2D^d^ and H2K^b^, respectively, than in MHC^−^ mice and in the other single MHC class I mice. A possible interpretation of these observations is that the frequency of “useful” NK cells in the peripheral NK cell repertoire is promoted during development, possibly by an influence on mechanisms regulatingcell survival, proliferation, or both.

When we scrutinized the interaction strengths between Ly49 receptors and MHC class I alleles predicted by the computer, it was clear that both models selected interaction strengths consistent with the binding properties that have been published (such as Ly49A-H2D^d^ and Ly49C-H2K^b^), even if the two-step selection model was more consistent in these particular cases. In addition, several more interactions were noted. Some of those could be explained by the simplifications included in the models. For example, one simplification is that the passing of selection is “all-or-none”, i.e. the cells either die or survive. *In vivo*, it is possible that “good” binding properties promotes proliferation and/or survival, but that also NK cells with less good self-MHC binding properties may pass selection and survive, although to a lesser extent. An extension of the model simulations, that included proliferation of selected cells, i.e., cells which express enough but not too many self MHC-specific receptors, and also a certain non-zero probability of escape from negative selection and export to the periphery of cells that did not pass the selection, also supported the two-step selection model above the sequential model. In fact, adding positive selection gave an even better fit to the data (Pickman *et al.*, *unpublished*). Results supporting the two-step selection model were also obtained in separate simulations that also included activating Ly49 receptors (Salmon-Divon *et al.*, *unpublished*). To explain subset overrepresentation, we favor the idea of proliferation, since cell death during NK cell development does not seem to be as massive as that of developing B and T cells (our unpublished data), and hence additional processes must be utilized to vary receptor expression frequencies in the post-education repertoire. A prediction from this notion is that those NK cells should incorporate BrdU more efficiently *in vivo*, in the presence of their ligands, compared to other NK cell subsets [25]. Further studies will be required to address this issue.

Yet another explanation for the unexpectedly large number of interactions, is that model construction was constrained by the available data. For example, the models create NK cell repertoires that are entirely “post-educational”, while the experimentally measured repertoires may also contain cells that have not yet completed all developmental stages. Additionally, only four receptors were included in the experiments and hence in the simulations. Thus, the complexity of the models, coupled to the intrinsic variability of the experimental data, may allow some additional, less realistic parameter value combinations to fit the data.

On the other hand, the mismatches between the modelling results and the literature could also reflect shortcomings of the *in vitro* assays used to determine Ly49/MHC class I binding patterns [31]–[34]. Binding assays using MHC class I multimers and cell-cell binding assays may underestimate low affinity interactions of physiological importance *in vivo*. It could also be that other factors that are different between the setups obscure the picture. Interpreted in this way, our model would reveal novel, biologically relevant Ly49/MHC class I interactions *in vivo* that have escaped detection due to sensitivity limitations of *in vitro* binding assays. Indeed, our functional analysis supported the modelling predictions by providing direct evidence indicating education by virtually all subsets and all MHC class I alleles tested. If this notion holds true, it may also shed light on the discrepancy between our conclusions and previous results from experiments with Ly49 receptor transgenic mice, taken to support the sequential model [12]. The latter data could be interpreted within the two-step selection model if additional binding properties of the studied Ly49 receptors were evoked [12]. It is also possible that forced expression of an Ly49 transgene may upset normal selection, contributing to the discrepant results between the studies.

Recent findings have shown that some NK cells in normal MHC class I-expressing mice and humans lack all known self-MHC–specific inhibitory receptors, yet are self-tolerant [24]–[26]. These cells exhibit a normal cell surface phenotype and some functional activity. However, they respond poorly to class I–deficient normal cells, tumor cells, or cross-linking of stimulatory receptors, suggesting that self-tolerance is established by insufficient stimulatory signalling. In this sense, these NK cells are similar to NK cells from MHC class I-deficient mice and humans [19], [35]–[37]. The possibility that NK cells could lack self-specific Ly49 receptors and still be allowed to mature is in our model at least partly covered by the addition of the “unknown receptor”, which was required for the models to work. Importantly, the fourth receptor may be regarded as a totality of all mechanisms by which NK cells failing Ly49-mediated selection may still survive. In fact, in many best-fit cases, a predominant role for the “unknown receptor” was found (Tables S1 and S2), suggesting an important role for a mechanism complementary to the Ly49/MHC class I interaction in our three-receptor simulations. NK cell subsets expressing no (known) self-specific receptors may have been educated through other mechanisms that do not affect the Ly49 repertoire, including adjustment of their ability to respond, a proposed mechanism in the process altering “cellular responsiveness” as a result of MHC education.

NK cell education may include a combination of several types of mechanisms, from negative (cell-death based) through positive (cell proliferation-based) selection to fine-tuning via adjustment of the cell's ability to respond to activating stimuli. An obvious mechanism for such fine-tuning would be the development of the hyporesponsive state, whether by “disarming” [38], failure to become “licensed” [39], or, as we prefer to call it, adjusting the “rheostat” of NK cell responsiveness [30], [40]. The possibility of other, non MHC-specific receptors contributing to the education process [41] should also be further investigated. Further studies and cellular markers for altered “cellular responsiveness” would be required to analyze the relative weights of all possible education mechanisms in generating the functional, post-education NK cell repertoire.

## Materials and Methods

### Repertoire simulations

Two computer models that simulated the educational steps according to either the sequential hypothesis or the two-step selection hypothesis had been created earlier [28], [29]. The two models were here modified to simulate education in single MHC class I mice, using three Ly49 receptor with known pre-education frequencies, and a fourth receptor with unknown pre-education frequency. The binding specificities of all receptors were varied between individual runs of the program. The models are shown schematically in Fig 1. Values of all the parameters used in the runs of the two models are listed in Table 1. The models are described more extensively in the results section.

### Mice

All manipulation of research animals were approved by a local committee for animal ethics appointed by the Swedish Governement. Handling of mice were onl done with scientists and staff with appropriate education and training. Mice were bred and maintained at the Department of Microbiology Tumor and Cell Biology (Karolinska Institutet, Stockholm, Sweden). C57BL/6 (B6) mice were originally obtained from Bomholt gård (Ry, Denmark). Mice carrying single H2K^b^ or single H2D^b^ MHC class I molecules were a kind gift from Francois Lemonnier and have been described [42]. Mice expressing H2D^d^ or H2L^d^ only have been described [18].

### Antibodies and flow cytometry

For FACS analysis in Fig. 1, splenocytes were isolated and NK cells were enriched by negative selection using the SpinSep mouse NK cell enrichment kit according to the manufacturer's protocol (Stemcell Technologies, Meylan, France). Fc receptors were blocked by incubation with 2.4G2 (anti FcγRIII) and remaining antibodies from the negative selection was blocked by incubation with rat serum. For FACS analysis, PK136-PerCPCy5.5 (NK1.1), anti-CD3-APC-Cy7, YLI-90-FITC/-PE (anti-Ly49I), 4D11-FITC/-APC (anti-Ly49G2), and streptavidin-PE were purchased from PharMingen/BD biosciences (Stockholm, Sweden). YE1/48 hybridoma (anti-Ly49A) was grown in our laboratory, purified and conjugated to Alexa-633 (Molecular Probes). 4LO3311-biotin (anti-Ly49C) was a kind gift from Suzanne Lemieux. Samples were collected on a FACSDiva flow cytometer and analyzed using the FACSDiva Software (BD biosciences, Stockholm, Sweden). Cells were gated on NK1.1^+^CD3^−^ lymphocytes. For nine color FACS (Fig. 3), the followign antibodies were used: anti NK1.1(PK136) PerCP-Cy5.5 (145-2C11), anti-Ly49G2(4D11)-APC, anti-Ly49F-PE (HBF-719), anti CD107a-FITC (1D4B), were purchased from BD PharMingen (Stockholm, Sweden). Anti-Ly49C (4LO3311) biotin and hybridoma was a kind gift from Suzanne Lemieux and was used together with anti-mouse IgG3-APC-Cy7 or PE-Cy7 (Southern Biotech). Anti-Ly49I purified (YLI-90, eBiosciences) was used with Zenon Kit Alexa700 (Invitrogen). Anti-Ly49A biotin (YE1/48, Stem Cell Technologies, Canada) was used with streptavidin QD565 (Invitrogen). NKG2A/C/E (20d5) purified (BD Biosciences) was conjugated to Pacific Blue using antibody labeling kit (Molecular Probes/Invitrogen. For functional experiments, dead cells were excluded using the Vivid near IR reagent (Invitrogen). Results were acquired with a FACSAria (BD Biosciences, Mountain View, USA) and analyzed using the FlowJo software (Three Star, Stanford, USA).

### In vitro stimulation assay

This assay was perfored essentially as previously reported [43], In brief, 1.5*10^6^ splenocytes from naïve mice were added to plates pre-coated with 50 µg/ml anti-NK1.1 (PK136), anti-NKG2D (A10) antibodies. Anti-CD107a (LAMP-1) was included during the stimulation (5 µg/ml) together with Monensin GolgiStop (BD Pharmingen) and 500 U/ml of IL-2. Plates were incubated at 37°C for 4–6 h followed by surface staining and FACS analysis.

### Fit of simulation results to experimental data

To identify and analyze simulations that gave rise to repertoires similar to the experimental ones, we fitted the computer-generated repertoires to the Ly49 frequencies obtained by experimentation. The experimental data from each staining included frequencies of cells expressing none, singles, pairs and triples of the three examined Ly49 receptors. Thus, each experiment provided eight data points to which the results of the simulation were fitted. The fit, ƒ, is defined as the RMS, that is, the root of the sum of the squared deviations of the simulated points, *S_j_*, from the experimental ones, *E_i,j_*, divided by the number of mice per group, *n* ,and by the number of experiential data points, which was 8.
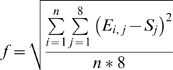
The smaller the fit, the closer the simulation results are to the experimental ones. Within each series of runs (for each MHC class I background and receptor staining), the runs with parameter values that gave the smallest fit values were considered to be the best-fit parameter value sets. We could not include the dependencies between receptor expression frequencies of single receptors, pairs and triplets in the calculations of significance of differences in fit values [44], because the data (four to six mice per group) were not sufficient to take the dependencies into account. However, the difference between the two examined models was already clearly evident at the RMS level, and hence finer statistical analysis was not necessary.

For each MHC class I background and receptor staining, we compared the best RMS values obtained by the two-model using the F-test, and – since 16 comparisons were made between the two models – we performed the FDR correction for multiple comparisons on the results of the F-test.

## Supporting Information

Table S1 (0.11 MB PDF)

Table S2 (0.10 MB PDF)
