# Supplementary material for: Probing Natural Killer Cell Education by Ly49 Receptor Expression Analysis and Computational Modelling in Single MHC Class I Mice
Source: PLoS One. 2009 Jun 25;4(6):e6046. doi: 10.1371/journal.pone.0006046 (PMC2699029; doi:10.1371/journal.pone.0006046)
Supplement: Table S1 — (0.11 MB PDF) [file pone.0006046.s001.pdf]

Supplemental Table S1. Parameters for the two-step selection model

**Ly49A,G2,C**

| **Kb** | **RMS** | **Smin** | **Smax** | **Ly49A** | **Ly49G2** | **Ly49C** | **4$^{th}$** strength | **4$^{th}$** prob | **4$^{th}$** expr |
|---|---|---|---|---|---|---|---|---|---|
| | 1,34751 | 3 | 7 | 2 | 2 | 3 | 4 | 0,45 | 0,6174759 |
| *Kb* | | | | *insuff* | *insuff* | *suff* | *suff* | | |
| **Db** | **RMS** | **Smin** | **Smax** | **Ly49A** | **Ly49G2** | **Ly49C** | **4$^{th}$** strength | **4$^{th}$** prob | **4$^{th}$** expr |
| | 2,48947 | 2 | 4 | 0 | 1 | 1 | 2 | 0,75 | 0,9478658 |
| | 2,48947 | 2 | 5 | 0 | 1 | 1 | 3 | 0,75 | 0,9478658 |
| | 2,48947 | 2 | 6 | 0 | 1 | 1 | 4 | 0,75 | 0,9478658 |
| | 2,48947 | 3 | 6 | 0 | 1 | 2 | 3 | 0,75 | 0,9478658 |
| | 2,48947 | 3 | 7 | 0 | 1 | 2 | 4 | 0,75 | 0,9478658 |
| | 2,48947 | 4 | 8 | 0 | 1 | 3 | 4 | 0,75 | 0,9478658 |
| | 2,48947 | 3 | 6 | 0 | 2 | 1 | 3 | 0,75 | 0,9478658 |
| | 2,48947 | 3 | 7 | 0 | 2 | 1 | 4 | 0,75 | 0,9478658 |
| | 2,48947 | 3 | 7 | 0 | 2 | 2 | 3 | 0,75 | 0,9478658 |
| | 2,48947 | 3 | 8 | 0 | 2 | 2 | 4 | 0,75 | 0,9478658 |
| | 2,48947 | 4 | 8 | 0 | 2 | 2 | 4 | 0,75 | 0,9478658 |
| | 2,48947 | 4 | 9 | 0 | 2 | 3 | 4 | 0,75 | 0,9478658 |
| | 2,48947 | 4 | 8 | 0 | 3 | 1 | 4 | 0,75 | 0,9478658 |
| | 2,48947 | 4 | 9 | 0 | 3 | 2 | 4 | 0,75 | 0,9478658 |
| | 2,48947 | 4 | 10 | 0 | 3 | 3 | 4 | 0,75 | 0,9478658 |
| | 2,48947 | 4 | 9 | 1 | 2 | 2 | 4 | 0,75 | 0,9478658 |
| *Db* | | | | *no/insuff* | *insuff* | *insuff* | *suff* | | |
| **Dd** | **RMS** | **Smin** | **Smax** | **Ly49A** | **Ly49G2** | **Ly49C** | **4$^{th}$** strength | **4$^{th}$** prob | **4$^{th}$** expr |
| | 2,32443 | 2 | 6 | 1 | 3 | 2 | 4 | 0,55 | 0,4308975 |
| *Dd* | | | | *insuff* | *suff* | *suff* | *suff* | | |
| **Ld** | **RMS** | **Smin** | **Smax** | **Ly49A** | **Ly49G2** | **Ly49C** | **4$^{th}$** strength | **4$^{th}$** prob | **4$^{th}$** expr |
| | 2,80068 | 1 | 3 | 2 | 0 | 1 | 2 | 0,65 | 0,776491 |
| | 2,80068 | 1 | 4 | 2 | 0 | 1 | 3 | 0,65 | 0,776491 |
| | 2,80068 | 1 | 5 | 2 | 0 | 1 | 4 | 0,65 | 0,776491 |
| | 2,80068 | 1 | 4 | 3 | 0 | 1 | 2 | 0,65 | 0,776491 |
| | 2,80068 | 1 | 4 | 3 | 0 | 1 | 3 | 0,65 | 0,776491 |
| | 2,80068 | 1 | 5 | 3 | 0 | 1 | 3 | 0,65 | 0,776491 |
| | 2,80068 | 1 | 5 | 3 | 0 | 1 | 4 | 0,65 | 0,776491 |
| | 2,80068 | 1 | 6 | 3 | 0 | 1 | 4 | 0,65 | 0,776491 |
| | 2,80068 | 1 | 5 | 3 | 0 | 2 | 3 | 0,65 | 0,776491 |
| | 2,80068 | 2 | 5 | 3 | 0 | 2 | 3 | 0,65 | 0,776491 |
| | 2,80068 | 1 | 6 | 3 | 0 | 2 | 4 | 0,65 | 0,776491 |
| | 2,80068 | 2 | 6 | 3 | 0 | 2 | 4 | 0,65 | 0,776491 |
| | 2,80068 | 1 | 5 | 4 | 0 | 1 | 2 | 0,65 | 0,776491 |
| | 2,80068 | 1 | 5 | 4 | 0 | 1 | 3 | 0,65 | 0,776491 |
| | 2,80068 | 1 | 6 | 4 | 0 | 1 | 3 | 0,65 | 0,776491 |
| | 2,80068 | 1 | 5 | 4 | 0 | 1 | 4 | 0,65 | 0,776491 |
| | 2,80068 | 1 | 6 | 4 | 0 | 1 | 4 | 0,65 | 0,776491 |

| | | | | | | | | | |
|---|---|---|---|---|---|---|---|---|---|
| | 2,80068 | 1 | 7 | 4 | 0 | 1 | 4 | 0,65 | 0,776491 |
| | 2,80068 | 1 | 6 | 4 | 0 | 2 | 3 | 0,65 | 0,776491 |
| | 2,80068 | 2 | 6 | 4 | 0 | 2 | 3 | 0,65 | 0,776491 |
| | 2,80068 | 1 | 6 | 4 | 0 | 2 | 4 | 0,65 | 0,776491 |

**Ly49A,G2,C *cont***

| **Ld** | **RMS** | **Smin** | **Smax** | **Ly49A** | **Ly49G2** | **Ly49C** | **4$^{th}$** strength | **4$^{th}$** prob | **4$^{th}$** expr |
|---|---|---|---|---|---|---|---|---|---|
| | 2,80068 | 1 | 7 | 4 | 0 | 2 | 4 | 0,65 | 0,776491 |
| | 2,80068 | 2 | 6 | 4 | 0 | 2 | 4 | 0,65 | 0,776491 |
| | 2,80068 | 2 | 7 | 4 | 0 | 2 | 4 | 0,65 | 0,776491 |
| | 2,80068 | 1 | 7 | 4 | 0 | 3 | 4 | 0,65 | 0,776491 |
| | 2,80068 | 2 | 7 | 4 | 0 | 3 | 4 | 0,65 | 0,776491 |
| | 2,80068 | 3 | 7 | 4 | 0 | 3 | 4 | 0,65 | 0,776491 |
| | 2,80068 | 2 | 7 | 4 | 1 | 2 | 4 | 0,65 | 0,776491 |
| *Ld* | | | | *suff* | *no/insuff* | *suff* | *suff* | | |

**Ly49A,G2,I**

| **Kb** | **RMS** | **Smin** | **Smax** | **Ly49A** | **Ly49G2** | **Ly49I** | **4$^{th}$** strength | **4$^{th}$** prob | **4$^{th}$** expr |
|---|---|---|---|---|---|---|---|---|---|
| | 2,03425 | 4 | 8 | 2 | 2 | 3 | 4 | 0,65 | 0,7126142 |
| *Kb* | | | | *insuff* | *insuff* | *insuff* | *suff* | | |
| **Db** | **RMS** | **Smin** | **Smax** | **Ly49A** | **Ly49G2** | **Ly49I** | **4$^{th}$** strength | **4$^{th}$** prob | **4$^{th}$** expr |
| | 2,82212 | 1 | 1 | 0 | 0 | 1 | 1 | 0,6 | 0,4576271 |
| | 2,82212 | 1 | 2 | 0 | 0 | 1 | 2 | 0,6 | 0,4576271 |
| | 2,82212 | 1 | 3 | 0 | 0 | 1 | 3 | 0,6 | 0,4576271 |
| | 2,82212 | 1 | 4 | 0 | 0 | 1 | 4 | 0,6 | 0,4576271 |
| | 2,82212 | 1 | 2 | 0 | 0 | 2 | 1 | 0,6 | 0,4576271 |
| | 2,82212 | 1 | 2 | 0 | 0 | 2 | 2 | 0,6 | 0,4576271 |
| | 2,82212 | 1 | 3 | 0 | 0 | 2 | 2 | 0,6 | 0,4576271 |
| | 2,82212 | 2 | 2 | 0 | 0 | 2 | 2 | 0,6 | 0,4576271 |
| | 2,82212 | 2 | 3 | 0 | 0 | 2 | 2 | 0,6 | 0,4576271 |
| | 2,82212 | 1 | 3 | 0 | 0 | 2 | 3 | 0,6 | 0,4576271 |
| | 2,82212 | 1 | 4 | 0 | 0 | 2 | 3 | 0,6 | 0,4576271 |
| | 2,82212 | 2 | 3 | 0 | 0 | 2 | 3 | 0,6 | 0,4576271 |
| | 2,82212 | 2 | 4 | 0 | 0 | 2 | 3 | 0,6 | 0,4576271 |
| | 2,82212 | 1 | 4 | 0 | 0 | 2 | 4 | 0,6 | 0,4576271 |
| | 2,82212 | 1 | 5 | 0 | 0 | 2 | 4 | 0,6 | 0,4576271 |
| | 2,82212 | 2 | 4 | 0 | 0 | 2 | 4 | 0,6 | 0,4576271 |
| | 2,82212 | 2 | 5 | 0 | 0 | 2 | 4 | 0,6 | 0,4576271 |
| | 2,82212 | 1 | 3 | 0 | 0 | 3 | 1 | 0,6 | 0,4576271 |
| | 2,82212 | 1 | 3 | 0 | 0 | 3 | 2 | 0,6 | 0,4576271 |
| | 2,82212 | 1 | 4 | 0 | 0 | 3 | 2 | 0,6 | 0,4576271 |
| | 2,82212 | 2 | 3 | 0 | 0 | 3 | 2 | 0,6 | 0,4576271 |
| | 2,82212 | 2 | 4 | 0 | 0 | 3 | 2 | 0,6 | 0,4576271 |
| | 2,82212 | 1 | 3 | 0 | 0 | 3 | 3 | 0,6 | 0,4576271 |
| | 2,82212 | 1 | 4 | 0 | 0 | 3 | 3 | 0,6 | 0,4576271 |

| | | | | | | | | |
|---|---|---|---|---|---|---|---|---|
| 2,82212 | 1 | 5 | 0 | 0 | 3 | 3 | 0,6 | 0,4576271 |
| 2,82212 | 2 | 3 | 0 | 0 | 3 | 3 | 0,6 | 0,4576271 |
| 2,82212 | 2 | 4 | 0 | 0 | 3 | 3 | 0,6 | 0,4576271 |
| 2,82212 | 2 | 5 | 0 | 0 | 3 | 3 | 0,6 | 0,4576271 |
| 2,82212 | 3 | 3 | 0 | 0 | 3 | 3 | 0,6 | 0,4576271 |
| 2,82212 | 3 | 4 | 0 | 0 | 3 | 3 | 0,6 | 0,4576271 |
| 2,82212 | 3 | 5 | 0 | 0 | 3 | 3 | 0,6 | 0,4576271 |
| 2,82212 | 1 | 4 | 0 | 0 | 3 | 4 | 0,6 | 0,4576271 |

**Ly49A,G2,I *cont***

| Db | RMS | Smin | Smax | Ly49A | Ly49G2 | Ly49I | 4th strength | 4th prob | 4th expr |
|---|---|---|---|---|---|---|---|---|---|
| | 2,82212 | 1 | 5 | 0 | 0 | 3 | 4 | 0,6 | 0,4576271 |
| | 2,82212 | 1 | 6 | 0 | 0 | 3 | 4 | 0,6 | 0,4576271 |
| | 2,82212 | 2 | 4 | 0 | 0 | 3 | 4 | 0,6 | 0,4576271 |
| | 2,82212 | 2 | 5 | 0 | 0 | 3 | 4 | 0,6 | 0,4576271 |
| | 2,82212 | 2 | 6 | 0 | 0 | 3 | 4 | 0,6 | 0,4576271 |
| | 2,82212 | 3 | 4 | 0 | 0 | 3 | 4 | 0,6 | 0,4576271 |
| | 2,82212 | 3 | 5 | 0 | 0 | 3 | 4 | 0,6 | 0,4576271 |
| | 2,82212 | 3 | 6 | 0 | 0 | 3 | 4 | 0,6 | 0,4576271 |
| | 2,82212 | 1 | 4 | 0 | 0 | 4 | 1 | 0,6 | 0,4576271 |
| | 2,82212 | 1 | 4 | 0 | 0 | 4 | 2 | 0,6 | 0,4576271 |
| | 2,82212 | 1 | 5 | 0 | 0 | 4 | 2 | 0,6 | 0,4576271 |
| | 2,82212 | 2 | 4 | 0 | 0 | 4 | 2 | 0,6 | 0,4576271 |
| | 2,82212 | 2 | 5 | 0 | 0 | 4 | 2 | 0,6 | 0,4576271 |
| | 2,82212 | 1 | 4 | 0 | 0 | 4 | 3 | 0,6 | 0,4576271 |
| | 2,82212 | 1 | 5 | 0 | 0 | 4 | 3 | 0,6 | 0,4576271 |
| | 2,82212 | 1 | 6 | 0 | 0 | 4 | 3 | 0,6 | 0,4576271 |
| | 2,82212 | 2 | 4 | 0 | 0 | 4 | 3 | 0,6 | 0,4576271 |
| | 2,82212 | 2 | 5 | 0 | 0 | 4 | 3 | 0,6 | 0,4576271 |
| | 2,82212 | 2 | 6 | 0 | 0 | 4 | 3 | 0,6 | 0,4576271 |
| | 2,82212 | 3 | 4 | 0 | 0 | 4 | 3 | 0,6 | 0,4576271 |
| | 2,82212 | 3 | 5 | 0 | 0 | 4 | 3 | 0,6 | 0,4576271 |
| | 2,82212 | 3 | 6 | 0 | 0 | 4 | 3 | 0,6 | 0,4576271 |
| | 2,82212 | 1 | 4 | 0 | 0 | 4 | 4 | 0,6 | 0,4576271 |
| | 2,82212 | 1 | 5 | 0 | 0 | 4 | 4 | 0,6 | 0,4576271 |
| | 2,82212 | 1 | 6 | 0 | 0 | 4 | 4 | 0,6 | 0,4576271 |
| | 2,82212 | 1 | 7 | 0 | 0 | 4 | 4 | 0,6 | 0,4576271 |
| | 2,82212 | 2 | 4 | 0 | 0 | 4 | 4 | 0,6 | 0,4576271 |
| | 2,82212 | 2 | 5 | 0 | 0 | 4 | 4 | 0,6 | 0,4576271 |
| | 2,82212 | 2 | 6 | 0 | 0 | 4 | 4 | 0,6 | 0,4576271 |
| | 2,82212 | 2 | 7 | 0 | 0 | 4 | 4 | 0,6 | 0,4576271 |
| | 2,82212 | 3 | 4 | 0 | 0 | 4 | 4 | 0,6 | 0,4576271 |
| | 2,82212 | 3 | 5 | 0 | 0 | 4 | 4 | 0,6 | 0,4576271 |
| | 2,82212 | 3 | 6 | 0 | 0 | 4 | 4 | 0,6 | 0,4576271 |
| | 2,82212 | 3 | 7 | 0 | 0 | 4 | 4 | 0,6 | 0,4576271 |
| | 2,82212 | 4 | 4 | 0 | 0 | 4 | 4 | 0,6 | 0,4576271 |

| | | | | | | | | | |
|---|---|---|---|---|---|---|---|---|---|
| | 2,82212 | 4 | 5 | 0 | 0 | 4 | 4 | 0,6 | 0,4576271 |
| | 2,82212 | 4 | 6 | 0 | 0 | 4 | 4 | 0,6 | 0,4576271 |
| | 2,82212 | 4 | 7 | 0 | 0 | 4 | 4 | 0,6 | 0,4576271 |
| | 2,82212 | 2 | 3 | 0 | 1 | 2 | 2 | 0,6 | 0,4576271 |
| | 2,82212 | 2 | 4 | 0 | 1 | 2 | 3 | 0,6 | 0,4576271 |
| | 2,82212 | 2 | 5 | 0 | 1 | 2 | 4 | 0,6 | 0,4576271 |
| | 2,82212 | 2 | 4 | 0 | 1 | 3 | 2 | 0,6 | 0,4576271 |
| | 2,82212 | 2 | 4 | 0 | 1 | 3 | 3 | 0,6 | 0,4576271 |
| | 2,82212 | 2 | 5 | 0 | 1 | 3 | 3 | 0,6 | 0,4576271 |
| | 2,82212 | 3 | 4 | 0 | 1 | 3 | 3 | 0,6 | 0,4576271 |
| | 2,82212 | 3 | 5 | 0 | 1 | 3 | 3 | 0,6 | 0,4576271 |

**Ly49A,G2,I *cont***

| Db | RMS | Smin | Smax | Ly49A | Ly49G2 | Ly49I | 4th strength | 4th prob | 4th expr |
|---|---|---|---|---|---|---|---|---|---|
| | 2,82212 | 2 | 5 | 0 | 1 | 3 | 4 | 0,6 | 0,4576271 |
| | 2,82212 | 2 | 6 | 0 | 1 | 3 | 4 | 0,6 | 0,4576271 |
| | 2,82212 | 3 | 5 | 0 | 1 | 3 | 4 | 0,6 | 0,4576271 |
| | 2,82212 | 3 | 6 | 0 | 1 | 3 | 4 | 0,6 | 0,4576271 |
| | 2,82212 | 2 | 5 | 0 | 1 | 4 | 2 | 0,6 | 0,4576271 |
| | 2,82212 | 2 | 5 | 0 | 1 | 4 | 3 | 0,6 | 0,4576271 |
| | 2,82212 | 2 | 6 | 0 | 1 | 4 | 3 | 0,6 | 0,4576271 |
| | 2,82212 | 3 | 5 | 0 | 1 | 4 | 3 | 0,6 | 0,4576271 |
| | 2,82212 | 3 | 6 | 0 | 1 | 4 | 3 | 0,6 | 0,4576271 |
| | 2,82212 | 2 | 5 | 0 | 1 | 4 | 4 | 0,6 | 0,4576271 |
| | 2,82212 | 2 | 6 | 0 | 1 | 4 | 4 | 0,6 | 0,4576271 |
| | 2,82212 | 2 | 7 | 0 | 1 | 4 | 4 | 0,6 | 0,4576271 |
| | 2,82212 | 3 | 5 | 0 | 1 | 4 | 4 | 0,6 | 0,4576271 |
| | 2,82212 | 3 | 6 | 0 | 1 | 4 | 4 | 0,6 | 0,4576271 |
| | 2,82212 | 3 | 7 | 0 | 1 | 4 | 4 | 0,6 | 0,4576271 |
| | 2,82212 | 4 | 5 | 0 | 1 | 4 | 4 | 0,6 | 0,4576271 |
| | 2,82212 | 4 | 6 | 0 | 1 | 4 | 4 | 0,6 | 0,4576271 |
| | 2,82212 | 4 | 7 | 0 | 1 | 4 | 4 | 0,6 | 0,4576271 |
| | 2,82212 | 3 | 5 | 0 | 2 | 3 | 3 | 0,6 | 0,4576271 |
| | 2,82212 | 3 | 6 | 0 | 2 | 3 | 4 | 0,6 | 0,4576271 |
| | 2,82212 | 3 | 6 | 0 | 2 | 4 | 3 | 0,6 | 0,4576271 |
| | 2,82212 | 3 | 6 | 0 | 2 | 4 | 4 | 0,6 | 0,4576271 |
| | 2,82212 | 3 | 7 | 0 | 2 | 4 | 4 | 0,6 | 0,4576271 |
| | 2,82212 | 4 | 6 | 0 | 2 | 4 | 4 | 0,6 | 0,4576271 |
| | 2,82212 | 4 | 7 | 0 | 2 | 4 | 4 | 0,6 | 0,4576271 |
| | 2,82212 | 4 | 7 | 0 | 3 | 4 | 4 | 0,6 | 0,4576271 |
| | 2,82212 | 2 | 3 | 1 | 0 | 2 | 2 | 0,6 | 0,4576271 |
| | 2,82212 | 2 | 4 | 1 | 0 | 2 | 3 | 0,6 | 0,4576271 |
| | 2,82212 | 2 | 5 | 1 | 0 | 2 | 4 | 0,6 | 0,4576271 |
| | 2,82212 | 2 | 4 | 1 | 0 | 3 | 2 | 0,6 | 0,4576271 |
| | 2,82212 | 2 | 4 | 1 | 0 | 3 | 3 | 0,6 | 0,4576271 |
| | 2,82212 | 2 | 5 | 1 | 0 | 3 | 3 | 0,6 | 0,4576271 |

| | | | | | | | | | |
|---|---|---|---|---|---|---|---|---|---|
| | 2,82212 | 3 | 4 | 1 | 0 | 3 | 3 | 0,6 | 0,4576271 |
| | 2,82212 | 3 | 5 | 1 | 0 | 3 | 3 | 0,6 | 0,4576271 |
| | 2,82212 | 2 | 5 | 1 | 0 | 3 | 4 | 0,6 | 0,4576271 |
| | 2,82212 | 2 | 6 | 1 | 0 | 3 | 4 | 0,6 | 0,4576271 |
| | 2,82212 | 3 | 5 | 1 | 0 | 3 | 4 | 0,6 | 0,4576271 |
| | 2,82212 | 3 | 6 | 1 | 0 | 3 | 4 | 0,6 | 0,4576271 |
| | 2,82212 | 2 | 5 | 1 | 0 | 4 | 2 | 0,6 | 0,4576271 |
| | 2,82212 | 2 | 5 | 1 | 0 | 4 | 3 | 0,6 | 0,4576271 |
| | 2,82212 | 2 | 6 | 1 | 0 | 4 | 3 | 0,6 | 0,4576271 |
| | 2,82212 | 3 | 5 | 1 | 0 | 4 | 3 | 0,6 | 0,4576271 |
| | 2,82212 | 3 | 6 | 1 | 0 | 4 | 3 | 0,6 | 0,4576271 |
| | 2,82212 | 2 | 5 | 1 | 0 | 4 | 4 | 0,6 | 0,4576271 |
| | 2,82212 | 2 | 6 | 1 | 0 | 4 | 4 | 0,6 | 0,4576271 |
| | 2,82212 | 2 | 7 | 1 | 0 | 4 | 4 | 0,6 | 0,4576271 |

**Ly49A,G2,I *cont***

| **Db** | **RMS** | **Smin** | **Smax** | **Ly49A** | **Ly49G2** | **Ly49I** | **4th strength** | **4th prob** | **4th expr** |
|---|---|---|---|---|---|---|---|---|---|
| | 2,82212 | 3 | 5 | 1 | 0 | 4 | 4 | 0,6 | 0,4576271 |
| | 2,82212 | 3 | 6 | 1 | 0 | 4 | 4 | 0,6 | 0,4576271 |
| | 2,82212 | 3 | 7 | 1 | 0 | 4 | 4 | 0,6 | 0,4576271 |
| | 2,82212 | 4 | 5 | 1 | 0 | 4 | 4 | 0,6 | 0,4576271 |
| | 2,82212 | 4 | 6 | 1 | 0 | 4 | 4 | 0,6 | 0,4576271 |
| | 2,82212 | 4 | 7 | 1 | 0 | 4 | 4 | 0,6 | 0,4576271 |
| | 2,82212 | 3 | 5 | 1 | 1 | 3 | 3 | 0,6 | 0,4576271 |
| | 2,82212 | 3 | 6 | 1 | 1 | 3 | 4 | 0,6 | 0,4576271 |
| | 2,82212 | 3 | 6 | 1 | 1 | 4 | 3 | 0,6 | 0,4576271 |
| | 2,82212 | 3 | 6 | 1 | 1 | 4 | 4 | 0,6 | 0,4576271 |
| | 2,82212 | 3 | 7 | 1 | 1 | 4 | 4 | 0,6 | 0,4576271 |
| | 2,82212 | 4 | 6 | 1 | 1 | 4 | 4 | 0,6 | 0,4576271 |
| | 2,82212 | 4 | 7 | 1 | 1 | 4 | 4 | 0,6 | 0,4576271 |
| | 2,82212 | 4 | 7 | 1 | 2 | 4 | 4 | 0,6 | 0,4576271 |
| | 2,82212 | 3 | 5 | 2 | 0 | 3 | 3 | 0,6 | 0,4576271 |
| | 2,82212 | 3 | 6 | 2 | 0 | 3 | 4 | 0,6 | 0,4576271 |
| | 2,82212 | 3 | 6 | 2 | 0 | 4 | 3 | 0,6 | 0,4576271 |
| | 2,82212 | 3 | 6 | 2 | 0 | 4 | 4 | 0,6 | 0,4576271 |
| | 2,82212 | 3 | 7 | 2 | 0 | 4 | 4 | 0,6 | 0,4576271 |
| | 2,82212 | 4 | 6 | 2 | 0 | 4 | 4 | 0,6 | 0,4576271 |
| | 2,82212 | 4 | 7 | 2 | 0 | 4 | 4 | 0,6 | 0,4576271 |
| | 2,82212 | 4 | 7 | 2 | 1 | 4 | 4 | 0,6 | 0,4576271 |
| | 2,82212 | 4 | 7 | 3 | 0 | 4 | 4 | 0,6 | 0,4576271 |
| *Db* | | | | *no/insuff* | *no/insuff* | *suff* | *suff* | | |

| **Dd** | **RMS** | **Smin** | **Smax** | **Ly49A** | **Ly49G2** | **Ly49I** | **4th strength** | **4th prob** | **4th expr** |
|---|---|---|---|---|---|---|---|---|---|
| | 2,61419 | 3 | 6 | 3 | 2 | 2 | 3 | 0,6 | 0,671233 |
| | 2,61419 | 4 | 8 | 4 | 2 | 3 | 4 | 0,6 | 0,671233 |
| | 2,61419 | 4 | 8 | 4 | 3 | 2 | 4 | 0,6 | 0,671233 |
| | 2,61419 | 4 | 8 | 4 | 3 | 3 | 4 | 0,6 | 0,671233 |

Supplemental Table S1. Parameters for the two-step selection model

| | | | | | | | | | |
|---|---|---|---|---|---|---|---|---|---|
| | 2,61419 | 4 | 9 | 4 | 3 | 3 | 4 | 0,6 | 0,671233 |
| *Dd* | | | | *suff* | *insuff* | *insuff* | *suff* | | |
| **Ld** | **RMS** | **Smin** | **Smax** | **Ly49A** | **Ly49G2** | **Ly49I** | **4**th strength | **4**th prob | **4**th expr |
| | 2,32593 | 2 | 6 | 3 | 2 | 1 | 3 | 0,8 | 0,8410696 |
| | 2,32593 | 2 | 7 | 3 | 2 | 1 | 4 | 0,8 | 0,8410696 |
| | 2,32593 | 2 | 7 | 4 | 2 | 1 | 3 | 0,8 | 0,8410696 |
| | 2,32593 | 2 | 8 | 4 | 2 | 1 | 4 | 0,8 | 0,8410696 |
| | 2,32593 | 2 | 8 | 4 | 3 | 1 | 4 | 0,8 | 0,8410696 |
| | 2,32593 | 3 | 8 | 4 | 3 | 1 | 4 | 0,8 | 0,8410696 |
| | 2,32593 | 3 | 9 | 4 | 3 | 2 | 4 | 0,8 | 0,8410696 |
| *Ld* | | | | *suff* | *suff* | *insuff* | *suff* | | |

Supplemental Table S1. Parameters for the two-step selection model

## Ly49A,I,C

| **Kb** | **RMS** | **Smin** | **Smax** | **Ly49A** | **Ly49I** | **Ly49C** | **4th strength** | **4th prob** | **4th expr** |
|---|---|---|---|---|---|---|---|---|---|
| | 2,56045 | 1 | 5 | 1 | 3 | 1 | 3 | 0,6 | 0,4042277 |
| | 2,56045 | 1 | 6 | 1 | 3 | 1 | 4 | 0,6 | 0,4042277 |
| | 2,56045 | 1 | 6 | 1 | 4 | 1 | 3 | 0,6 | 0,4042277 |
| | 2,56045 | 1 | 6 | 1 | 4 | 1 | 4 | 0,6 | 0,4042277 |
| | 2,56045 | 1 | 7 | 1 | 4 | 1 | 4 | 0,6 | 0,4042277 |
| | 2,56045 | 1 | 7 | 1 | 4 | 2 | 4 | 0,6 | 0,4042277 |
| | 2,56045 | 1 | 7 | 2 | 4 | 1 | 4 | 0,6 | 0,4042277 |
| *Kb* | | | | *suff* | *suff* | *suff* | *suff* | | |
| **Db** | **RMS** | **Smin** | **Smax** | **Ly49A** | **Ly49I** | **Ly49C** | **4th strength** | **4th prob** | **4th expr** |
| | 2,51254 | 1 | 1 | 0 | 1 | 0 | 1 | 0,6 | 0,4576271 |
| | 2,51254 | 1 | 2 | 0 | 1 | 0 | 2 | 0,6 | 0,4576271 |
| | 2,51254 | 1 | 3 | 0 | 1 | 0 | 3 | 0,6 | 0,4576271 |
| | 2,51254 | 1 | 4 | 0 | 1 | 0 | 4 | 0,6 | 0,4576271 |
| | 2,51254 | 1 | 2 | 0 | 2 | 0 | 1 | 0,6 | 0,4576271 |
| | 2,51254 | 1 | 2 | 0 | 2 | 0 | 2 | 0,6 | 0,4576271 |
| | 2,51254 | 1 | 3 | 0 | 2 | 0 | 2 | 0,6 | 0,4576271 |
| | 2,51254 | 2 | 2 | 0 | 2 | 0 | 2 | 0,6 | 0,4576271 |
| | 2,51254 | 2 | 3 | 0 | 2 | 0 | 2 | 0,6 | 0,4576271 |
| | 2,51254 | 1 | 3 | 0 | 2 | 0 | 3 | 0,6 | 0,4576271 |
| | 2,51254 | 1 | 4 | 0 | 2 | 0 | 3 | 0,6 | 0,4576271 |
| | 2,51254 | 2 | 3 | 0 | 2 | 0 | 3 | 0,6 | 0,4576271 |
| | 2,51254 | 2 | 4 | 0 | 2 | 0 | 3 | 0,6 | 0,4576271 |
| | 2,51254 | 1 | 4 | 0 | 2 | 0 | 4 | 0,6 | 0,4576271 |
| | 2,51254 | 1 | 5 | 0 | 2 | 0 | 4 | 0,6 | 0,4576271 |
| | 2,51254 | 2 | 4 | 0 | 2 | 0 | 4 | 0,6 | 0,4576271 |
| | 2,51254 | 2 | 5 | 0 | 2 | 0 | 4 | 0,6 | 0,4576271 |
| | 2,51254 | 2 | 3 | 0 | 2 | 1 | 2 | 0,6 | 0,4576271 |
| | 2,51254 | 2 | 4 | 0 | 2 | 1 | 3 | 0,6 | 0,4576271 |
| | 2,51254 | 2 | 5 | 0 | 2 | 1 | 4 | 0,6 | 0,4576271 |
| | 2,51254 | 1 | 3 | 0 | 3 | 0 | 1 | 0,6 | 0,4576271 |
| | 2,51254 | 1 | 3 | 0 | 3 | 0 | 2 | 0,6 | 0,4576271 |
| | 2,51254 | 1 | 4 | 0 | 3 | 0 | 2 | 0,6 | 0,4576271 |
| | 2,51254 | 2 | 3 | 0 | 3 | 0 | 2 | 0,6 | 0,4576271 |
| | 2,51254 | 2 | 4 | 0 | 3 | 0 | 2 | 0,6 | 0,4576271 |
| | 2,51254 | 1 | 3 | 0 | 3 | 0 | 3 | 0,6 | 0,4576271 |
| | 2,51254 | 1 | 4 | 0 | 3 | 0 | 3 | 0,6 | 0,4576271 |
| | 2,51254 | 1 | 5 | 0 | 3 | 0 | 3 | 0,6 | 0,4576271 |
| | 2,51254 | 2 | 3 | 0 | 3 | 0 | 3 | 0,6 | 0,4576271 |
| | 2,51254 | 2 | 4 | 0 | 3 | 0 | 3 | 0,6 | 0,4576271 |
| | 2,51254 | 2 | 5 | 0 | 3 | 0 | 3 | 0,6 | 0,4576271 |
| | 2,51254 | 3 | 3 | 0 | 3 | 0 | 3 | 0,6 | 0,4576271 |
| | 2,51254 | 3 | 4 | 0 | 3 | 0 | 3 | 0,6 | 0,4576271 |

| | | | | | | | | | |
|---|---|---|---|---|---|---|---|---|---|
| | 2,51254 | 3 | 5 | 0 | 3 | 0 | 3 | 0,6 | 0,4576271 |
| | 2,51254 | 1 | 4 | 0 | 3 | 0 | 4 | 0,6 | 0,4576271 |
| | 2,51254 | 1 | 5 | 0 | 3 | 0 | 4 | 0,6 | 0,4576271 |

**Ly49A,I,C *cont***

| Db | RMS | Smin | Smax | Ly49A | Ly49I | Ly49C | 4th strength | 4th prob | 4th expr |
|---|---|---|---|---|---|---|---|---|---|
| | 2,51254 | 1 | 6 | 0 | 3 | 0 | 4 | 0,6 | 0,4576271 |
| | 2,51254 | 2 | 4 | 0 | 3 | 0 | 4 | 0,6 | 0,4576271 |
| | 2,51254 | 2 | 5 | 0 | 3 | 0 | 4 | 0,6 | 0,4576271 |
| | 2,51254 | 2 | 6 | 0 | 3 | 0 | 4 | 0,6 | 0,4576271 |
| | 2,51254 | 3 | 4 | 0 | 3 | 0 | 4 | 0,6 | 0,4576271 |
| | 2,51254 | 3 | 5 | 0 | 3 | 0 | 4 | 0,6 | 0,4576271 |
| | 2,51254 | 3 | 6 | 0 | 3 | 0 | 4 | 0,6 | 0,4576271 |
| | 2,51254 | 2 | 4 | 0 | 3 | 1 | 2 | 0,6 | 0,4576271 |
| | 2,51254 | 2 | 4 | 0 | 3 | 1 | 3 | 0,6 | 0,4576271 |
| | 2,51254 | 2 | 5 | 0 | 3 | 1 | 3 | 0,6 | 0,4576271 |
| | 2,51254 | 3 | 4 | 0 | 3 | 1 | 3 | 0,6 | 0,4576271 |
| | 2,51254 | 3 | 5 | 0 | 3 | 1 | 3 | 0,6 | 0,4576271 |
| | 2,51254 | 2 | 5 | 0 | 3 | 1 | 4 | 0,6 | 0,4576271 |
| | 2,51254 | 2 | 6 | 0 | 3 | 1 | 4 | 0,6 | 0,4576271 |
| | 2,51254 | 3 | 5 | 0 | 3 | 1 | 4 | 0,6 | 0,4576271 |
| | 2,51254 | 3 | 6 | 0 | 3 | 1 | 4 | 0,6 | 0,4576271 |
| | 2,51254 | 3 | 5 | 0 | 3 | 2 | 3 | 0,6 | 0,4576271 |
| | 2,51254 | 3 | 6 | 0 | 3 | 2 | 4 | 0,6 | 0,4576271 |
| | 2,51254 | 1 | 4 | 0 | 4 | 0 | 1 | 0,6 | 0,4576271 |
| | 2,51254 | 1 | 4 | 0 | 4 | 0 | 2 | 0,6 | 0,4576271 |
| | 2,51254 | 1 | 5 | 0 | 4 | 0 | 2 | 0,6 | 0,4576271 |
| | 2,51254 | 2 | 4 | 0 | 4 | 0 | 2 | 0,6 | 0,4576271 |
| | 2,51254 | 2 | 5 | 0 | 4 | 0 | 2 | 0,6 | 0,4576271 |
| | 2,51254 | 1 | 4 | 0 | 4 | 0 | 3 | 0,6 | 0,4576271 |
| | 2,51254 | 1 | 5 | 0 | 4 | 0 | 3 | 0,6 | 0,4576271 |
| | 2,51254 | 1 | 6 | 0 | 4 | 0 | 3 | 0,6 | 0,4576271 |
| | 2,51254 | 2 | 4 | 0 | 4 | 0 | 3 | 0,6 | 0,4576271 |
| | 2,51254 | 2 | 5 | 0 | 4 | 0 | 3 | 0,6 | 0,4576271 |
| | 2,51254 | 2 | 6 | 0 | 4 | 0 | 3 | 0,6 | 0,4576271 |
| | 2,51254 | 3 | 4 | 0 | 4 | 0 | 3 | 0,6 | 0,4576271 |
| | 2,51254 | 3 | 5 | 0 | 4 | 0 | 3 | 0,6 | 0,4576271 |
| | 2,51254 | 3 | 6 | 0 | 4 | 0 | 3 | 0,6 | 0,4576271 |
| | 2,51254 | 1 | 4 | 0 | 4 | 0 | 4 | 0,6 | 0,4576271 |
| | 2,51254 | 1 | 5 | 0 | 4 | 0 | 4 | 0,6 | 0,4576271 |
| | 2,51254 | 1 | 6 | 0 | 4 | 0 | 4 | 0,6 | 0,4576271 |
| | 2,51254 | 1 | 7 | 0 | 4 | 0 | 4 | 0,6 | 0,4576271 |
| | 2,51254 | 2 | 4 | 0 | 4 | 0 | 4 | 0,6 | 0,4576271 |
| | 2,51254 | 2 | 5 | 0 | 4 | 0 | 4 | 0,6 | 0,4576271 |
| | 2,51254 | 2 | 6 | 0 | 4 | 0 | 4 | 0,6 | 0,4576271 |
| | 2,51254 | 2 | 7 | 0 | 4 | 0 | 4 | 0,6 | 0,4576271 |

| | | | | | | | | |
|---|---|---|---|---|---|---|---|---|
| 2,51254 | 3 | 4 | 0 | 4 | 0 | 4 | 0,6 | 0,4576271 |
| 2,51254 | 3 | 5 | 0 | 4 | 0 | 4 | 0,6 | 0,4576271 |
| 2,51254 | 3 | 6 | 0 | 4 | 0 | 4 | 0,6 | 0,4576271 |
| 2,51254 | 3 | 7 | 0 | 4 | 0 | 4 | 0,6 | 0,4576271 |
| 2,51254 | 4 | 4 | 0 | 4 | 0 | 4 | 0,6 | 0,4576271 |
| 2,51254 | 4 | 5 | 0 | 4 | 0 | 4 | 0,6 | 0,4576271 |

**Ly49A,I,C *cont***

| Db | RMS | Smin | Smax | Ly49A | Ly49I | Ly49C | 4th strength | 4th prob | 4th expr |
|---|---|---|---|---|---|---|---|---|---|
| | 2,51254 | 4 | 6 | 0 | 4 | 0 | 4 | 0,6 | 0,4576271 |
| | 2,51254 | 4 | 7 | 0 | 4 | 0 | 4 | 0,6 | 0,4576271 |
| | 2,51254 | 2 | 5 | 0 | 4 | 1 | 2 | 0,6 | 0,4576271 |
| | 2,51254 | 2 | 5 | 0 | 4 | 1 | 3 | 0,6 | 0,4576271 |
| | 2,51254 | 2 | 6 | 0 | 4 | 1 | 3 | 0,6 | 0,4576271 |
| | 2,51254 | 3 | 5 | 0 | 4 | 1 | 3 | 0,6 | 0,4576271 |
| | 2,51254 | 3 | 6 | 0 | 4 | 1 | 3 | 0,6 | 0,4576271 |
| | 2,51254 | 2 | 5 | 0 | 4 | 1 | 4 | 0,6 | 0,4576271 |
| | 2,51254 | 2 | 6 | 0 | 4 | 1 | 4 | 0,6 | 0,4576271 |
| | 2,51254 | 2 | 7 | 0 | 4 | 1 | 4 | 0,6 | 0,4576271 |
| | 2,51254 | 3 | 5 | 0 | 4 | 1 | 4 | 0,6 | 0,4576271 |
| | 2,51254 | 3 | 6 | 0 | 4 | 1 | 4 | 0,6 | 0,4576271 |
| | 2,51254 | 3 | 7 | 0 | 4 | 1 | 4 | 0,6 | 0,4576271 |
| | 2,51254 | 4 | 5 | 0 | 4 | 1 | 4 | 0,6 | 0,4576271 |
| | 2,51254 | 4 | 6 | 0 | 4 | 1 | 4 | 0,6 | 0,4576271 |
| | 2,51254 | 4 | 7 | 0 | 4 | 1 | 4 | 0,6 | 0,4576271 |
| | 2,51254 | 3 | 6 | 0 | 4 | 2 | 3 | 0,6 | 0,4576271 |
| | 2,51254 | 3 | 6 | 0 | 4 | 2 | 4 | 0,6 | 0,4576271 |
| | 2,51254 | 3 | 7 | 0 | 4 | 2 | 4 | 0,6 | 0,4576271 |
| | 2,51254 | 4 | 6 | 0 | 4 | 2 | 4 | 0,6 | 0,4576271 |
| | 2,51254 | 4 | 7 | 0 | 4 | 2 | 4 | 0,6 | 0,4576271 |
| | 2,51254 | 4 | 7 | 0 | 4 | 3 | 4 | 0,6 | 0,4576271 |
| | 2,51254 | 2 | 3 | 1 | 2 | 0 | 2 | 0,6 | 0,4576271 |
| | 2,51254 | 2 | 4 | 1 | 2 | 0 | 3 | 0,6 | 0,4576271 |
| | 2,51254 | 2 | 5 | 1 | 2 | 0 | 4 | 0,6 | 0,4576271 |
| | 2,51254 | 2 | 4 | 1 | 3 | 0 | 2 | 0,6 | 0,4576271 |
| | 2,51254 | 2 | 4 | 1 | 3 | 0 | 3 | 0,6 | 0,4576271 |
| | 2,51254 | 2 | 5 | 1 | 3 | 0 | 3 | 0,6 | 0,4576271 |
| | 2,51254 | 3 | 4 | 1 | 3 | 0 | 3 | 0,6 | 0,4576271 |
| | 2,51254 | 3 | 5 | 1 | 3 | 0 | 3 | 0,6 | 0,4576271 |
| | 2,51254 | 2 | 5 | 1 | 3 | 0 | 4 | 0,6 | 0,4576271 |
| | 2,51254 | 2 | 6 | 1 | 3 | 0 | 4 | 0,6 | 0,4576271 |
| | 2,51254 | 3 | 5 | 1 | 3 | 0 | 4 | 0,6 | 0,4576271 |
| | 2,51254 | 3 | 6 | 1 | 3 | 0 | 4 | 0,6 | 0,4576271 |
| | 2,51254 | 3 | 5 | 1 | 3 | 1 | 3 | 0,6 | 0,4576271 |
| | 2,51254 | 3 | 6 | 1 | 3 | 1 | 4 | 0,6 | 0,4576271 |
| | 2,51254 | 2 | 5 | 1 | 4 | 0 | 2 | 0,6 | 0,4576271 |

| | | | | | | | | | |
|---|---|---|---|---|---|---|---|---|---|
| | 2,51254 | 2 | 5 | 1 | 4 | 0 | 3 | 0,6 | 0,4576271 |
| | 2,51254 | 2 | 6 | 1 | 4 | 0 | 3 | 0,6 | 0,4576271 |
| | 2,51254 | 3 | 5 | 1 | 4 | 0 | 3 | 0,6 | 0,4576271 |
| | 2,51254 | 3 | 6 | 1 | 4 | 0 | 3 | 0,6 | 0,4576271 |
| | 2,51254 | 2 | 5 | 1 | 4 | 0 | 4 | 0,6 | 0,4576271 |
| | 2,51254 | 2 | 6 | 1 | 4 | 0 | 4 | 0,6 | 0,4576271 |
| | 2,51254 | 2 | 7 | 1 | 4 | 0 | 4 | 0,6 | 0,4576271 |
| | 2,51254 | 3 | 5 | 1 | 4 | 0 | 4 | 0,6 | 0,4576271 |
| | 2,51254 | 3 | 6 | 1 | 4 | 0 | 4 | 0,6 | 0,4576271 |

**Ly49A,I,C *cont***

| **Db** | **RMS** | **Smin** | **Smax** | **Ly49A** | **Ly49I** | **Ly49C** | **4$^{th}$ strength** | **4$^{th}$ prob** | **4$^{th}$ expr** |
|---|---|---|---|---|---|---|---|---|---|
| | 2,51254 | 3 | 7 | 1 | 4 | 0 | 4 | 0,6 | 0,4576271 |
| | 2,51254 | 4 | 5 | 1 | 4 | 0 | 4 | 0,6 | 0,4576271 |
| | 2,51254 | 4 | 6 | 1 | 4 | 0 | 4 | 0,6 | 0,4576271 |
| | 2,51254 | 4 | 7 | 1 | 4 | 0 | 4 | 0,6 | 0,4576271 |
| | 2,51254 | 3 | 6 | 1 | 4 | 1 | 3 | 0,6 | 0,4576271 |
| | 2,51254 | 3 | 6 | 1 | 4 | 1 | 4 | 0,6 | 0,4576271 |
| | 2,51254 | 3 | 7 | 1 | 4 | 1 | 4 | 0,6 | 0,4576271 |
| | 2,51254 | 4 | 6 | 1 | 4 | 1 | 4 | 0,6 | 0,4576271 |
| | 2,51254 | 4 | 7 | 1 | 4 | 1 | 4 | 0,6 | 0,4576271 |
| | 2,51254 | 4 | 7 | 1 | 4 | 2 | 4 | 0,6 | 0,4576271 |
| | 2,51254 | 3 | 5 | 2 | 3 | 0 | 3 | 0,6 | 0,4576271 |
| | 2,51254 | 3 | 6 | 2 | 3 | 0 | 4 | 0,6 | 0,4576271 |
| | 2,51254 | 3 | 6 | 2 | 4 | 0 | 3 | 0,6 | 0,4576271 |
| | 2,51254 | 3 | 6 | 2 | 4 | 0 | 4 | 0,6 | 0,4576271 |
| | 2,51254 | 3 | 7 | 2 | 4 | 0 | 4 | 0,6 | 0,4576271 |
| | 2,51254 | 4 | 6 | 2 | 4 | 0 | 4 | 0,6 | 0,4576271 |
| | 2,51254 | 4 | 7 | 2 | 4 | 0 | 4 | 0,6 | 0,4576271 |
| | 2,51254 | 4 | 7 | 2 | 4 | 1 | 4 | 0,6 | 0,4576271 |
| | 2,51254 | 4 | 7 | 3 | 4 | 0 | 4 | 0,6 | 0,4576271 |
| *Db* | | | | *insuff* | *suff* | *no* | *suff* | | |
| **Dd** | RMS | Smin | Smax | Ly49A | Ly49I | Ly49C | **4$^{th}$ strength** | **4$^{th}$ prob** | **4$^{th}$ expr** |
| | 3,12833 | 2 | 6 | 3 | 1 | 2 | 3 | 0,65 | 0,7863008 |
| | 3,12833 | 2 | 7 | 3 | 1 | 2 | 4 | 0,65 | 0,7863008 |
| | 3,12833 | 2 | 7 | 4 | 1 | 2 | 3 | 0,65 | 0,7863008 |
| | 3,12833 | 2 | 8 | 4 | 1 | 2 | 4 | 0,65 | 0,7863008 |
| | 3,12833 | 2 | 8 | 4 | 1 | 3 | 4 | 0,65 | 0,7863008 |
| | 3,12833 | 3 | 8 | 4 | 1 | 3 | 4 | 0,65 | 0,7863008 |
| | 3,12833 | 3 | 9 | 4 | 2 | 3 | 4 | 0,65 | 0,7863008 |
| *Dd* | | | | *suff* | *insuff* | *suff* | *suff* | | |
| **Ld** | **RMS** | **Smin** | **Smax** | **Ly49A** | **Ly49I** | **Ly49C** | **4$^{th}$ strength** | **4$^{th}$ prob** | **4$^{th}$ expr** |
| | 2,33259 | 1 | 3 | 2 | 0 | 1 | 2 | 0,65 | 0,7764911 |
| | 2,33259 | 1 | 4 | 2 | 0 | 1 | 3 | 0,65 | 0,7764911 |
| | 2,33259 | 1 | 5 | 2 | 0 | 1 | 4 | 0,65 | 0,7764911 |

| | | | | | | | | | |
|---|---|---|---|---|---|---|---|---|---|
| | 2,33259 | 1 | 4 | 3 | 0 | 1 | 2 | 0,65 | 0,7764911 |
| | 2,33259 | 1 | 4 | 3 | 0 | 1 | 3 | 0,65 | 0,7764911 |
| | 2,33259 | 1 | 5 | 3 | 0 | 1 | 3 | 0,65 | 0,7764911 |
| | 2,33259 | 1 | 5 | 3 | 0 | 1 | 4 | 0,65 | 0,7764911 |
| | 2,33259 | 1 | 6 | 3 | 0 | 1 | 4 | 0,65 | 0,7764911 |
| | 2,33259 | 1 | 5 | 3 | 0 | 2 | 3 | 0,65 | 0,7764911 |
| | 2,33259 | 2 | 5 | 3 | 0 | 2 | 3 | 0,65 | 0,7764911 |
| | 2,33259 | 1 | 6 | 3 | 0 | 2 | 4 | 0,65 | 0,7764911 |
| | 2,33259 | 2 | 6 | 3 | 0 | 2 | 4 | 0,65 | 0,7764911 |
| | 2,33259 | 1 | 5 | 4 | 0 | 1 | 2 | 0,65 | 0,7764911 |
| | 2,33259 | 1 | 5 | 4 | 0 | 1 | 3 | 0,65 | 0,7764911 |
| | 2,33259 | 1 | 6 | 4 | 0 | 1 | 3 | 0,65 | 0,7764911 |
| | 2,33259 | 1 | 5 | 4 | 0 | 1 | 4 | 0,65 | 0,7764911 |

**Ly49A,I,C *cont***

| **Ld** | **RMS** | **Smin** | **Smax** | **Ly49A** | **Ly49I** | **Ly49C** | **4th** strength | **4th** prob | **4th** expr |
|---|---|---|---|---|---|---|---|---|---|
| | 2,33259 | 1 | 6 | 4 | 0 | 1 | 4 | 0,65 | 0,7764911 |
| | 2,33259 | 1 | 7 | 4 | 0 | 1 | 4 | 0,65 | 0,7764911 |
| | 2,33259 | 1 | 6 | 4 | 0 | 2 | 3 | 0,65 | 0,7764911 |
| | 2,33259 | 2 | 6 | 4 | 0 | 2 | 3 | 0,65 | 0,7764911 |
| | 2,33259 | 1 | 6 | 4 | 0 | 2 | 4 | 0,65 | 0,7764911 |
| | 2,33259 | 1 | 7 | 4 | 0 | 2 | 4 | 0,65 | 0,7764911 |
| | 2,33259 | 2 | 6 | 4 | 0 | 2 | 4 | 0,65 | 0,7764911 |
| | 2,33259 | 2 | 7 | 4 | 0 | 2 | 4 | 0,65 | 0,7764911 |
| | 2,33259 | 1 | 7 | 4 | 0 | 3 | 4 | 0,65 | 0,7764911 |
| | 2,33259 | 2 | 7 | 4 | 0 | 3 | 4 | 0,65 | 0,7764911 |
| | 2,33259 | 3 | 7 | 4 | 0 | 3 | 4 | 0,65 | 0,7764911 |
| | 2,33259 | 2 | 7 | 4 | 1 | 2 | 4 | 0,65 | 0,7764911 |
| *Ld* | | | | *suff* | *no* | *suff* | *suff* | | |

**Ly49G2,I,C**

| **Kb** | **RMS** | **Smin** | **Smax** | **Ly49G2** | **Ly49I** | **Ly49C** | **4th** strength | **4th** prob | **4th** expr |
|---|---|---|---|---|---|---|---|---|---|
| | 2,7552 | 1 | 5 | 1 | 3 | 1 | 3 | 0,55 | 0,3316755 |
| | 2,7552 | 1 | 6 | 1 | 3 | 1 | 4 | 0,55 | 0,3316755 |
| | 2,7552 | 1 | 6 | 1 | 4 | 1 | 3 | 0,55 | 0,3316755 |
| | 2,7552 | 1 | 6 | 1 | 4 | 1 | 4 | 0,55 | 0,3316755 |
| | 2,7552 | 1 | 7 | 1 | 4 | 1 | 4 | 0,55 | 0,3316755 |
| | 2,7552 | 1 | 7 | 1 | 4 | 2 | 4 | 0,55 | 0,3316755 |
| | 2,7552 | 1 | 7 | 2 | 4 | 1 | 4 | 0,55 | 0,3316755 |
| *Kb* | *Kb* | | | *suff* | *suff* | *suff* | *suff* | | |
| **Db** | **RMS** | **Smin** | **Smax** | **Ly49G2** | **Ly49I** | **Ly49C** | **4th** strength | **4th** prob | **4th** expr |
| | 2,53723 | 1 | 1 | 0 | 1 | 0 | 1 | 0,6 | 0,4576272 |
| | 2,53723 | 1 | 2 | 0 | 1 | 0 | 2 | 0,6 | 0,4576272 |
| | 2,53723 | 1 | 3 | 0 | 1 | 0 | 3 | 0,6 | 0,4576272 |
| | 2,53723 | 1 | 4 | 0 | 1 | 0 | 4 | 0,6 | 0,4576272 |

| | | | | | | | | | |
|---|---|---|---|---|---|---|---|---|---|
| | 2,53723 | 1 | 2 | 0 | 2 | 0 | 1 | 0,6 | 0,4576272 |
| | 2,53723 | 1 | 2 | 0 | 2 | 0 | 2 | 0,6 | 0,4576272 |
| | 2,53723 | 1 | 3 | 0 | 2 | 0 | 2 | 0,6 | 0,4576272 |
| | 2,53723 | 2 | 2 | 0 | 2 | 0 | 2 | 0,6 | 0,4576272 |
| | 2,53723 | 2 | 3 | 0 | 2 | 0 | 2 | 0,6 | 0,4576272 |
| | 2,53723 | 1 | 3 | 0 | 2 | 0 | 3 | 0,6 | 0,4576272 |
| | 2,53723 | 1 | 4 | 0 | 2 | 0 | 3 | 0,6 | 0,4576272 |
| | 2,53723 | 2 | 3 | 0 | 2 | 0 | 3 | 0,6 | 0,4576272 |
| | 2,53723 | 2 | 4 | 0 | 2 | 0 | 3 | 0,6 | 0,4576272 |
| | 2,53723 | 1 | 4 | 0 | 2 | 0 | 4 | 0,6 | 0,4576272 |
| | 2,53723 | 1 | 5 | 0 | 2 | 0 | 4 | 0,6 | 0,4576272 |
| | 2,53723 | 2 | 4 | 0 | 2 | 0 | 4 | 0,6 | 0,4576272 |
| | 2,53723 | 2 | 5 | 0 | 2 | 0 | 4 | 0,6 | 0,4576272 |
| | 2,53723 | 2 | 3 | 0 | 2 | 1 | 2 | 0,6 | 0,4576272 |
| | 2,53723 | 2 | 4 | 0 | 2 | 1 | 3 | 0,6 | 0,4576272 |
| | 2,53723 | 2 | 5 | 0 | 2 | 1 | 4 | 0,6 | 0,4576272 |
| | 2,53723 | 1 | 3 | 0 | 3 | 0 | 1 | 0,6 | 0,4576272 |

**Ly49G2,I,C *cont***

| Db | RMS | Smin | Smax | Ly49G2 | Ly49I | Ly49C | 4th strength | 4th prob | 4th expr |
|---|---|---|---|---|---|---|---|---|---|
| | 2,53723 | 1 | 3 | 0 | 3 | 0 | 2 | 0,6 | 0,4576272 |
| | 2,53723 | 1 | 4 | 0 | 3 | 0 | 2 | 0,6 | 0,4576272 |
| | 2,53723 | 2 | 3 | 0 | 3 | 0 | 2 | 0,6 | 0,4576272 |
| | 2,53723 | 2 | 4 | 0 | 3 | 0 | 2 | 0,6 | 0,4576272 |
| | 2,53723 | 1 | 3 | 0 | 3 | 0 | 3 | 0,6 | 0,4576272 |
| | 2,53723 | 1 | 4 | 0 | 3 | 0 | 3 | 0,6 | 0,4576272 |
| | 2,53723 | 1 | 5 | 0 | 3 | 0 | 3 | 0,6 | 0,4576272 |
| | 2,53723 | 2 | 3 | 0 | 3 | 0 | 3 | 0,6 | 0,4576272 |
| | 2,53723 | 2 | 4 | 0 | 3 | 0 | 3 | 0,6 | 0,4576272 |
| | 2,53723 | 2 | 5 | 0 | 3 | 0 | 3 | 0,6 | 0,4576272 |
| | 2,53723 | 3 | 3 | 0 | 3 | 0 | 3 | 0,6 | 0,4576272 |
| | 2,53723 | 3 | 4 | 0 | 3 | 0 | 3 | 0,6 | 0,4576272 |
| | 2,53723 | 3 | 5 | 0 | 3 | 0 | 3 | 0,6 | 0,4576272 |
| | 2,53723 | 1 | 4 | 0 | 3 | 0 | 4 | 0,6 | 0,4576272 |
| | 2,53723 | 1 | 5 | 0 | 3 | 0 | 4 | 0,6 | 0,4576272 |
| | 2,53723 | 1 | 6 | 0 | 3 | 0 | 4 | 0,6 | 0,4576272 |
| | 2,53723 | 2 | 4 | 0 | 3 | 0 | 4 | 0,6 | 0,4576272 |
| | 2,53723 | 2 | 5 | 0 | 3 | 0 | 4 | 0,6 | 0,4576272 |
| | 2,53723 | 2 | 6 | 0 | 3 | 0 | 4 | 0,6 | 0,4576272 |
| | 2,53723 | 3 | 4 | 0 | 3 | 0 | 4 | 0,6 | 0,4576272 |
| | 2,53723 | 3 | 5 | 0 | 3 | 0 | 4 | 0,6 | 0,4576272 |
| | 2,53723 | 3 | 6 | 0 | 3 | 0 | 4 | 0,6 | 0,4576272 |
| | 2,53723 | 2 | 4 | 0 | 3 | 1 | 2 | 0,6 | 0,4576272 |
| | 2,53723 | 2 | 4 | 0 | 3 | 1 | 3 | 0,6 | 0,4576272 |
| | 2,53723 | 2 | 5 | 0 | 3 | 1 | 3 | 0,6 | 0,4576272 |
| | 2,53723 | 3 | 4 | 0 | 3 | 1 | 3 | 0,6 | 0,4576272 |

| | | | | | | | | | |
|---|---|---|---|---|---|---|---|---|---|
| | 2,53723 | 3 | 5 | 0 | 3 | 1 | 3 | 0,6 | 0,4576272 |
| | 2,53723 | 2 | 5 | 0 | 3 | 1 | 4 | 0,6 | 0,4576272 |
| | 2,53723 | 2 | 6 | 0 | 3 | 1 | 4 | 0,6 | 0,4576272 |
| | 2,53723 | 3 | 5 | 0 | 3 | 1 | 4 | 0,6 | 0,4576272 |
| | 2,53723 | 3 | 6 | 0 | 3 | 1 | 4 | 0,6 | 0,4576272 |
| | 2,53723 | 3 | 5 | 0 | 3 | 2 | 3 | 0,6 | 0,4576272 |
| | 2,53723 | 3 | 6 | 0 | 3 | 2 | 4 | 0,6 | 0,4576272 |
| | 2,53723 | 1 | 4 | 0 | 4 | 0 | 1 | 0,6 | 0,4576272 |
| | 2,53723 | 1 | 4 | 0 | 4 | 0 | 2 | 0,6 | 0,4576272 |
| | 2,53723 | 1 | 5 | 0 | 4 | 0 | 2 | 0,6 | 0,4576272 |
| | 2,53723 | 2 | 4 | 0 | 4 | 0 | 2 | 0,6 | 0,4576272 |
| | 2,53723 | 2 | 5 | 0 | 4 | 0 | 2 | 0,6 | 0,4576272 |
| | 2,53723 | 1 | 4 | 0 | 4 | 0 | 3 | 0,6 | 0,4576272 |
| | 2,53723 | 1 | 5 | 0 | 4 | 0 | 3 | 0,6 | 0,4576272 |
| | 2,53723 | 1 | 6 | 0 | 4 | 0 | 3 | 0,6 | 0,4576272 |
| | 2,53723 | 2 | 4 | 0 | 4 | 0 | 3 | 0,6 | 0,4576272 |
| | 2,53723 | 2 | 5 | 0 | 4 | 0 | 3 | 0,6 | 0,4576272 |
| | 2,53723 | 2 | 6 | 0 | 4 | 0 | 3 | 0,6 | 0,4576272 |
| | 2,53723 | 3 | 4 | 0 | 4 | 0 | 3 | 0,6 | 0,4576272 |
| | 2,53723 | 3 | 5 | 0 | 4 | 0 | 3 | 0,6 | 0,4576272 |

**Ly49G2,I,C *cont***

| **Db** | **RMS** | **Smin** | **Smax** | **Ly49G2** | **Ly49I** | **Ly49C** | **$4^{th}$ strength** | **$4^{th}$ prob** | **$4^{th}$ expr** |
|---|---|---|---|---|---|---|---|---|---|
| | 2,53723 | 3 | 6 | 0 | 4 | 0 | 3 | 0,6 | 0,4576272 |
| | 2,53723 | 1 | 4 | 0 | 4 | 0 | 4 | 0,6 | 0,4576272 |
| | 2,53723 | 1 | 5 | 0 | 4 | 0 | 4 | 0,6 | 0,4576272 |
| | 2,53723 | 1 | 6 | 0 | 4 | 0 | 4 | 0,6 | 0,4576272 |
| | 2,53723 | 1 | 7 | 0 | 4 | 0 | 4 | 0,6 | 0,4576272 |
| | 2,53723 | 2 | 4 | 0 | 4 | 0 | 4 | 0,6 | 0,4576272 |
| | 2,53723 | 2 | 5 | 0 | 4 | 0 | 4 | 0,6 | 0,4576272 |
| | 2,53723 | 2 | 6 | 0 | 4 | 0 | 4 | 0,6 | 0,4576272 |
| | 2,53723 | 2 | 7 | 0 | 4 | 0 | 4 | 0,6 | 0,4576272 |
| | 2,53723 | 3 | 4 | 0 | 4 | 0 | 4 | 0,6 | 0,4576272 |
| | 2,53723 | 3 | 5 | 0 | 4 | 0 | 4 | 0,6 | 0,4576272 |
| | 2,53723 | 3 | 6 | 0 | 4 | 0 | 4 | 0,6 | 0,4576272 |
| | 2,53723 | 3 | 7 | 0 | 4 | 0 | 4 | 0,6 | 0,4576272 |
| | 2,53723 | 4 | 4 | 0 | 4 | 0 | 4 | 0,6 | 0,4576272 |
| | 2,53723 | 4 | 5 | 0 | 4 | 0 | 4 | 0,6 | 0,4576272 |
| | 2,53723 | 4 | 6 | 0 | 4 | 0 | 4 | 0,6 | 0,4576272 |
| | 2,53723 | 4 | 7 | 0 | 4 | 0 | 4 | 0,6 | 0,4576272 |
| | 2,53723 | 2 | 5 | 0 | 4 | 1 | 2 | 0,6 | 0,4576272 |
| | 2,53723 | 2 | 5 | 0 | 4 | 1 | 3 | 0,6 | 0,4576272 |
| | 2,53723 | 2 | 6 | 0 | 4 | 1 | 3 | 0,6 | 0,4576272 |
| | 2,53723 | 3 | 5 | 0 | 4 | 1 | 3 | 0,6 | 0,4576272 |
| | 2,53723 | 3 | 6 | 0 | 4 | 1 | 3 | 0,6 | 0,4576272 |
| | 2,53723 | 2 | 5 | 0 | 4 | 1 | 4 | 0,6 | 0,4576272 |

| | | | | | | | | | |
|---|---|---|---|---|---|---|---|---|---|
| | 2,53723 | 2 | 6 | 0 | 4 | 1 | 4 | 0,6 | 0,4576272 |
| | 2,53723 | 2 | 7 | 0 | 4 | 1 | 4 | 0,6 | 0,4576272 |
| | 2,53723 | 3 | 5 | 0 | 4 | 1 | 4 | 0,6 | 0,4576272 |
| | 2,53723 | 3 | 6 | 0 | 4 | 1 | 4 | 0,6 | 0,4576272 |
| | 2,53723 | 3 | 7 | 0 | 4 | 1 | 4 | 0,6 | 0,4576272 |
| | 2,53723 | 4 | 5 | 0 | 4 | 1 | 4 | 0,6 | 0,4576272 |
| | 2,53723 | 4 | 6 | 0 | 4 | 1 | 4 | 0,6 | 0,4576272 |
| | 2,53723 | 4 | 7 | 0 | 4 | 1 | 4 | 0,6 | 0,4576272 |
| | 2,53723 | 3 | 6 | 0 | 4 | 2 | 3 | 0,6 | 0,4576272 |
| | 2,53723 | 3 | 6 | 0 | 4 | 2 | 4 | 0,6 | 0,4576272 |
| | 2,53723 | 3 | 7 | 0 | 4 | 2 | 4 | 0,6 | 0,4576272 |
| | 2,53723 | 4 | 6 | 0 | 4 | 2 | 4 | 0,6 | 0,4576272 |
| | 2,53723 | 4 | 7 | 0 | 4 | 2 | 4 | 0,6 | 0,4576272 |
| | 2,53723 | 4 | 7 | 0 | 4 | 3 | 4 | 0,6 | 0,4576272 |
| | 2,53723 | 2 | 3 | 1 | 2 | 0 | 2 | 0,6 | 0,4576272 |
| | 2,53723 | 2 | 4 | 1 | 2 | 0 | 3 | 0,6 | 0,4576272 |
| | 2,53723 | 2 | 5 | 1 | 2 | 0 | 4 | 0,6 | 0,4576272 |
| | 2,53723 | 2 | 4 | 1 | 3 | 0 | 2 | 0,6 | 0,4576272 |
| | 2,53723 | 2 | 4 | 1 | 3 | 0 | 3 | 0,6 | 0,4576272 |
| | 2,53723 | 2 | 5 | 1 | 3 | 0 | 3 | 0,6 | 0,4576272 |
| | 2,53723 | 3 | 4 | 1 | 3 | 0 | 3 | 0,6 | 0,4576272 |
| | 2,53723 | 3 | 5 | 1 | 3 | 0 | 3 | 0,6 | 0,4576272 |
| | 2,53723 | 2 | 5 | 1 | 3 | 0 | 4 | 0,6 | 0,4576272 |

## Ly49G2,I,C *cont*

| Db | RMS | Smin | Smax | Ly49G2 | Ly49I | Ly49C | $4^{th}$ strength | $4^{th}$ prob | $4^{th}$ expr |
|---|---|---|---|---|---|---|---|---|---|
| | 2,53723 | 2 | 6 | 1 | 3 | 0 | 4 | 0,6 | 0,4576272 |
| | 2,53723 | 3 | 5 | 1 | 3 | 0 | 4 | 0,6 | 0,4576272 |
| | 2,53723 | 3 | 6 | 1 | 3 | 0 | 4 | 0,6 | 0,4576272 |
| | 2,53723 | 3 | 5 | 1 | 3 | 1 | 3 | 0,6 | 0,4576272 |
| | 2,53723 | 3 | 6 | 1 | 3 | 1 | 4 | 0,6 | 0,4576272 |
| | 2,53723 | 2 | 5 | 1 | 4 | 0 | 2 | 0,6 | 0,4576272 |
| | 2,53723 | 2 | 5 | 1 | 4 | 0 | 3 | 0,6 | 0,4576272 |
| | 2,53723 | 2 | 6 | 1 | 4 | 0 | 3 | 0,6 | 0,4576272 |
| | 2,53723 | 3 | 5 | 1 | 4 | 0 | 3 | 0,6 | 0,4576272 |
| | 2,53723 | 3 | 6 | 1 | 4 | 0 | 3 | 0,6 | 0,4576272 |
| | 2,53723 | 2 | 5 | 1 | 4 | 0 | 4 | 0,6 | 0,4576272 |
| | 2,53723 | 2 | 6 | 1 | 4 | 0 | 4 | 0,6 | 0,4576272 |
| | 2,53723 | 2 | 7 | 1 | 4 | 0 | 4 | 0,6 | 0,4576272 |
| | 2,53723 | 3 | 5 | 1 | 4 | 0 | 4 | 0,6 | 0,4576272 |
| | 2,53723 | 3 | 6 | 1 | 4 | 0 | 4 | 0,6 | 0,4576272 |
| | 2,53723 | 3 | 7 | 1 | 4 | 0 | 4 | 0,6 | 0,4576272 |
| | 2,53723 | 4 | 5 | 1 | 4 | 0 | 4 | 0,6 | 0,4576272 |
| | 2,53723 | 4 | 6 | 1 | 4 | 0 | 4 | 0,6 | 0,4576272 |
| | 2,53723 | 4 | 7 | 1 | 4 | 0 | 4 | 0,6 | 0,4576272 |
| | 2,53723 | 3 | 6 | 1 | 4 | 1 | 3 | 0,6 | 0,4576272 |

| | | | | | | | | | |
|---|---|---|---|---|---|---|---|---|---|
| | 2,53723 | 3 | 6 | 1 | 4 | 1 | 4 | 0,6 | 0,4576272 |
| | 2,53723 | 3 | 7 | 1 | 4 | 1 | 4 | 0,6 | 0,4576272 |
| | 2,53723 | 4 | 6 | 1 | 4 | 1 | 4 | 0,6 | 0,4576272 |
| | 2,53723 | 4 | 7 | 1 | 4 | 1 | 4 | 0,6 | 0,4576272 |
| | 2,53723 | 4 | 7 | 1 | 4 | 2 | 4 | 0,6 | 0,4576272 |
| | 2,53723 | 3 | 5 | 2 | 3 | 0 | 3 | 0,6 | 0,4576272 |
| | 2,53723 | 3 | 6 | 2 | 3 | 0 | 4 | 0,6 | 0,4576272 |
| | 2,53723 | 3 | 6 | 2 | 4 | 0 | 3 | 0,6 | 0,4576272 |
| | 2,53723 | 3 | 6 | 2 | 4 | 0 | 4 | 0,6 | 0,4576272 |
| | 2,53723 | 3 | 7 | 2 | 4 | 0 | 4 | 0,6 | 0,4576272 |
| | 2,53723 | 4 | 6 | 2 | 4 | 0 | 4 | 0,6 | 0,4576272 |
| | 2,53723 | 4 | 7 | 2 | 4 | 0 | 4 | 0,6 | 0,4576272 |
| | 2,53723 | 4 | 7 | 2 | 4 | 1 | 4 | 0,6 | 0,4576272 |
| | 2,53723 | 4 | 7 | 3 | 4 | 0 | 4 | 0,6 | 0,4576272 |
| *Db* | | | | *no/insuff* | *suff* | *no/insuff* | *suff* | | |
| **Dd** | **RMS** | **Smin** | **Smax** | **Ly49G2** | **Ly49I** | **Ly49C** | **4**th strength | **4**th prob | **4**th expr |
| | 2,52555 | 4 | 8 | 2 | 3 | 1 | 4 | 0,6 | 0,6891659 |
| *Dd* | | | | *W* | *W* | *W* | *S* | | |
| **Ld** | **RMS** | **Smin** | **Smax** | **Ly49G2** | **Ly49I** | **Ly49C** | **4**th strength | **4**th prob | **4**th expr |
| | 2,05446 | 3 | 7 | 1 | 1 | 2 | 3 | 0,65 | 0,8807707 |
| | 2,05446 | 3 | 8 | 1 | 1 | 2 | 4 | 0,65 | 0,8807707 |
| | 2,05446 | 4 | 9 | 1 | 1 | 3 | 4 | 0,65 | 0,8807707 |
| | 2,05446 | 4 | 10 | 1 | 2 | 3 | 4 | 0,65 | 0,8807707 |
| | 2,05446 | 4 | 10 | 2 | 1 | 3 | 4 | 0,65 | 0,8807707 |
| *Ld* | | | | *insuff* | *insuff* | *insuff* | *suff* | | |
