# Supplementary material for: Probing Natural Killer Cell Education by Ly49 Receptor Expression Analysis and Computational Modelling in Single MHC Class I Mice
Source: PLoS One. 2009 Jun 25;4(6):e6046. doi: 10.1371/journal.pone.0006046 (PMC2699029; doi:10.1371/journal.pone.0006046)
Supplement: Table S2 — (0.10 MB PDF) [file pone.0006046.s002.pdf]

Supplemental Table S2. Parameters for the sequential selection model

Ly49A,G2,C

Kb	RMS	Smin	Ly49A	Ly49G2	Ly49C	4th strength	4th prob	4th expr
	3,67119	1	0	0	1	1	0,4	0,57
	3,67119	2	0	0	2	2	0,4	0,57
	3,67119	2	0	1	2	2	0,4	0,57
	3,67119	2	1	0	2	2	0,4	0,57
	3,67119	3	0	0	3	3	0,4	0,57
	3,67119	3	0	1	3	3	0,4	0,57
	3,67119	3	0	2	3	3	0,4	0,57
	3,67119	3	1	0	3	3	0,4	0,57
	3,67119	3	1	1	3	3	0,4	0,57
	3,67119	3	2	0	3	3	0,4	0,57
	3,67119	4	0	0	4	4	0,4	0,57
	3,67119	4	0	1	4	4	0,4	0,57
	3,67119	4	0	2	4	4	0,4	0,57
	3,67119	4	0	3	4	4	0,4	0,57
	3,67119	4	1	0	4	4	0,4	0,57
	3,67119	4	1	1	4	4	0,4	0,57
	3,67119	4	1	2	4	4	0,4	0,57
	3,67119	4	2	0	4	4	0,4	0,57
	3,67119	4	2	1	4	4	0,4	0,57
	3,67119	4	3	0	4	4	0,4	0,57
Kb			*no/insuff*	*no/insuff*	*suff*	*suff*		

Db	RMS	Smin	Ly49A	Ly49G2	Ly49C	4th strength	4th prob	4th expr
	5,19752	1	0	1	0	1	0,4	0,42
	5,19752	2	0	2	0	2	0,4	0,42
	5,19752	2	1	2	0	2	0,4	0,42
	5,19752	2	0	2	1	2	0,4	0,42
	5,19752	3	0	3	0	3	0,4	0,42
	5,19752	3	1	3	0	3	0,4	0,42
	5,19752	3	2	3	0	3	0,4	0,42
	5,19752	3	0	3	1	3	0,4	0,42
	5,19752	3	1	3	1	3	0,4	0,42
	5,19752	3	0	3	2	3	0,4	0,42
	5,19752	4	0	4	0	4	0,4	0,42
	5,19752	4	1	4	0	4	0,4	0,42
	5,19752	4	2	4	0	4	0,4	0,42
	5,19752	4	3	4	0	4	0,4	0,42
	5,19752	4	0	4	1	4	0,4	0,42
	5,19752	4	1	4	1	4	0,4	0,42
	5,19752	4	2	4	1	4	0,4	0,42
	5,19752	4	0	4	2	4	0,4	0,42
	5,19752	4	1	4	2	4	0,4	0,42
	5,19752	4	0	4	3	4	0,4	0,42

Db			*no/insuff*	*suff*	*no/insuff*	*suff*		

Ly49A,G2,C cont

**Dd**	**RMS**	**Smin**	**Ly49A**	**Ly49G2**	**Ly49C**	**4th** strength	**4th** prob	**4th** expr
	4,42395	1	0	0	1	1	0,4	0,57
	4,42395	2	0	0	2	2	0,4	0,57
	4,42395	2	0	1	2	2	0,4	0,57
	4,42395	2	1	0	2	2	0,4	0,57
	4,42395	3	0	0	3	3	0,4	0,57
	4,42395	3	0	1	3	3	0,4	0,57
	4,42395	3	0	2	3	3	0,4	0,57
	4,42395	3	1	0	3	3	0,4	0,57
	4,42395	3	1	1	3	3	0,4	0,57
	4,42395	3	2	0	3	3	0,4	0,57
	4,42395	4	0	0	4	4	0,4	0,57
	4,42395	4	0	1	4	4	0,4	0,57
	4,42395	4	0	2	4	4	0,4	0,57
	4,42395	4	0	3	4	4	0,4	0,57
	4,42395	4	1	0	4	4	0,4	0,57
	4,42395	4	1	1	4	4	0,4	0,57
	4,42395	4	1	2	4	4	0,4	0,57
	4,42395	4	2	0	4	4	0,4	0,57
	4,42395	4	2	1	4	4	0,4	0,57
	4,42395	4	3	0	4	4	0,4	0,57
Dd			*no/insuff*	*no/insuff*	*suff*	*suff*		

**Ld**	**RMS**	**Smin**	**Ly49A**	**Ly49G2**	**Ly49C**	**4th** strength	**4th** prob	**4th** expr
	6,05438	1	0	1	0	1	0,3	0,36
	6,05438	2	0	2	0	2	0,3	0,36
	6,05438	2	1	2	0	2	0,3	0,36
	6,05438	2	0	2	1	2	0,3	0,36
	6,05438	3	0	3	0	3	0,3	0,36
	6,05438	3	1	3	0	3	0,3	0,36
	6,05438	3	2	3	0	3	0,3	0,36
	6,05438	3	0	3	1	3	0,3	0,36
	6,05438	3	1	3	1	3	0,3	0,36
	6,05438	3	0	3	2	3	0,3	0,36
	6,05438	4	0	4	0	4	0,3	0,36
	6,05438	4	1	4	0	4	0,3	0,36
	6,05438	4	2	4	0	4	0,3	0,36
	6,05438	4	3	4	0	4	0,3	0,36
	6,05438	4	0	4	1	4	0,3	0,36
	6,05438	4	1	4	1	4	0,3	0,36
	6,05438	4	2	4	1	4	0,3	0,36

	6,05438	4	0	4	2	4	0,3	0,36
	6,05438	4	1	4	2	4	0,3	0,36
	6,05438	4	0	4	3	4	0,3	0,36
Ld			*no/insuff*	*suff*	*no/insuff*	*suff*		

Ly49A,G2,I

**Kb**	**RMS**	**Smin**	**Ly49A**	**Ly49G2**	**Ly49I**	4th strength	4th prob	4th expr
	6,10778	2	1	1	2	2	0,4	0,35
	6,10778	3	1	2	3	3	0,4	0,35
	6,10778	3	2	1	3	3	0,4	0,35
	6,10778	3	2	2	3	3	0,4	0,35
	6,10778	4	1	3	4	4	0,4	0,35
	6,10778	4	2	2	4	4	0,4	0,35
	6,10778	4	2	3	4	4	0,4	0,35
	6,10778	4	3	1	4	4	0,4	0,35
	6,10778	4	3	2	4	4	0,4	0,35
	6,10778	4	3	3	4	4	0,4	0,35
Kb			*insuff*	*insuff*	*suff*	*suff*		
**Db**	**RMS**	**Smin**	**Ly49A**	**Ly49G2**	**Ly49I**	4th strength	4th prob	4th expr
	6,18668	1	0	1	0	1	0,3	0,36
	6,18668	2	0	2	0	2	0,3	0,36
	6,18668	2	1	2	0	2	0,3	0,36
	6,18668	2	0	2	1	2	0,3	0,36
	6,18668	3	0	3	0	3	0,3	0,36
	6,18668	3	1	3	0	3	0,3	0,36
	6,18668	3	2	3	0	3	0,3	0,36
	6,18668	3	0	3	1	3	0,3	0,36
	6,18668	3	1	3	1	3	0,3	0,36
	6,18668	3	0	3	2	3	0,3	0,36
	6,18668	4	0	4	0	4	0,3	0,36
	6,18668	4	1	4	0	4	0,3	0,36
	6,18668	4	2	4	0	4	0,3	0,36
	6,18668	4	3	4	0	4	0,3	0,36
	6,18668	4	0	4	1	4	0,3	0,36
	6,18668	4	1	4	1	4	0,3	0,36
	6,18668	4	2	4	1	4	0,3	0,36
	6,18668	4	0	4	2	4	0,3	0,36
	6,18668	4	1	4	2	4	0,3	0,36
	6,18668	4	0	4	3	4	0,3	0,36
Db			*no/insuff*	*suff*	*no/insuff*	*suff*		
**Dd**	**RMS**	**Smin**	**Ly49A**	**Ly49G2**	**Ly49I**	4th strength	4th prob	4th expr
	6,42074	3	1	2	1	2	0,6	0,64
	6,42074	4	1	3	1	3	0,6	0,64

6,42074	4	2	3	1	3	0,6	0,64
6,42074	4	1	3	2	3	0,6	0,64
6,42074	5	1	4	1	4	0,6	0,64
6,42074	5	2	4	1	4	0,6	0,64
6,42074	5	3	4	1	4	0,6	0,64
6,42074	5	1	4	2	4	0,6	0,64
6,42074	5	2	3	2	3	0,6	0,64
6,42074	5	2	3	2	4	0,6	0,64
6,42074	5	2	4	2	3	0,6	0,64

Ly49A,G2,I cont

**Dd**	**RMS**	**Smin**	**Ly49A**	**Ly49G2**	**Ly49I**	**4th** strength	**4th** prob	**4th** expr
	6,42074	5	2	4	2	4	0,6	0,64
	6,42074	5	1	4	3	4	0,6	0,64
	6,42074	6	2	4	2	4	0,6	0,64
	6,42074	6	3	4	2	4	0,6	0,64
	6,42074	6	2	4	3	4	0,6	0,64
	6,42074	7	3	4	3	4	0,6	0,64
Dd			*insuff*	*insuff*	*insuff*	*insuff*		

**Ld**	**RMS**	**Smin**	**Ly49A**	**Ly49G2**	**Ly49I**	**4th** strength	**4th** prob	**4th** expr
	5,16635	3	1	2	1	2	0,4	0,52
	5,16635	4	1	3	1	3	0,4	0,52
	5,16635	4	2	3	1	3	0,4	0,52
	5,16635	4	1	3	2	3	0,4	0,52
	5,16635	5	1	4	1	4	0,4	0,52
	5,16635	5	2	4	1	4	0,4	0,52
	5,16635	5	3	4	1	4	0,4	0,52
	5,16635	5	1	4	2	4	0,4	0,52
	5,16635	5	2	3	2	3	0,4	0,52
	5,16635	5	2	3	2	4	0,4	0,52
	5,16635	5	2	4	2	3	0,4	0,52
	5,16635	5	2	4	2	4	0,4	0,52
	5,16635	5	1	4	3	4	0,4	0,52
	5,16635	6	2	4	2	4	0,4	0,52
	5,16635	6	3	4	2	4	0,4	0,52
	5,16635	6	2	4	3	4	0,4	0,52
	5,16635	7	3	4	3	4	0,4	0,52
Ld			*insuff*	*insuff*	*insuff*	*insuff*		

Ly49A,I,C

**Kb**	**RMS**	**Smin**	**Ly49A**	**Ly49I**	**Ly49C**	**4th** strength	**4th** prob	**4th** expr
	4,64641	1	0	0	1	1	0,4	0,57
	4,64641	2	0	0	2	2	0,4	0,57
	4,64641	2	0	1	2	2	0,4	0,57
	4,64641	2	1	0	2	2	0,4	0,57

	4,64641	3	0	0	3	3	0,4	0,57
	4,64641	3	0	1	3	3	0,4	0,57
	4,64641	3	0	2	3	3	0,4	0,57
	4,64641	3	1	0	3	3	0,4	0,57
	4,64641	3	1	1	3	3	0,4	0,57
	4,64641	3	2	0	3	3	0,4	0,57
	4,64641	4	0	0	4	4	0,4	0,57
	4,64641	4	0	1	4	4	0,4	0,57
	4,64641	4	0	2	4	4	0,4	0,57
	4,64641	4	0	3	4	4	0,4	0,57
	4,64641	4	1	0	4	4	0,4	0,57
	4,64641	4	1	1	4	4	0,4	0,57

Ly49A,I,C cont

**Kb**	**RMS**	**Smin**	**Ly49A**	**Ly49I**	**Ly49C**	**4th** strength	**4th** prob	**4th** expr
	4,64641	4	1	2	4	4	0,4	0,57
	4,64641	4	2	0	4	4	0,4	0,57
	4,64641	4	2	1	4	4	0,4	0,57
	4,64641	4	3	0	4	4	0,4	0,57
Kb			*no/insuff*	*no/insuff*	*suff*	*suff*		
**Db**	**RMS**	**Smin**	**Ly49A**	**Ly49I**	**Ly49C**	**4th** strength	**4th** prob	**4th** expr
	5,14451	3	1	1	2	3	0,5	0,69
	5,14451	4	1	1	3	4	0,5	0,69
	5,14451	4	1	2	3	4	0,5	0,69
	5,14451	4	2	1	3	4	0,5	0,69
Db			*insuff*	*suff*	*no*	*suff*		
**Dd**	RMS	**Smin**	Ly49A	Ly49I	Ly49C	**4th** strength	**4th** prob	**4th** expr
	5,70129	2	2	1	1	2	0,5	0,57
	5,70129	3	3	2	1	3	0,5	0,57
	5,70129	3	3	1	2	3	0,5	0,57
	5,70129	3	3	2	2	3	0,5	0,57
	5,70129	4	4	3	1	4	0,5	0,57
	5,70129	4	4	2	2	4	0,5	0,57
	5,70129	4	4	3	2	4	0,5	0,57
	5,70129	4	4	1	3	4	0,5	0,57
	5,70129	4	4	2	3	4	0,5	0,57
	5,70129	4	4	3	3	4	0,5	0,57
Dd			*suff*	*insuff*	*insuff*	*suff*		
**Ld**	**RMS**	**Smin**	**Ly49A**	**Ly49I**	**Ly49C**	**4th** strength	**4th** prob	**4th** expr
	6,6784	1	0	1	0	1	0,3	0,33
	6,6784	2	0	2	0	2	0,3	0,33
	6,6784	2	1	2	0	2	0,3	0,33
	6,6784	2	0	2	1	2	0,3	0,33
	6,6784	3	0	3	0	3	0,3	0,33
	6,6784	3	1	3	0	3	0,3	0,33

	6,6784	3	2	3	0	3	0,3	0,33
	6,6784	3	0	3	1	3	0,3	0,33
	6,6784	3	1	3	1	3	0,3	0,33
	6,6784	3	0	3	2	3	0,3	0,33
	6,6784	4	0	4	0	4	0,3	0,33
	6,6784	4	1	4	0	4	0,3	0,33
	6,6784	4	2	4	0	4	0,3	0,33
	6,6784	4	3	4	0	4	0,3	0,33
	6,6784	4	0	4	1	4	0,3	0,33
	6,6784	4	1	4	1	4	0,3	0,33
	6,6784	4	2	4	1	4	0,3	0,33
	6,6784	4	0	4	2	4	0,3	0,33
	6,6784	4	1	4	2	4	0,3	0,33
	6,6784	4	0	4	3	4	0,3	0,33
Ld			*no/insuff*	*suff*	*no/insuff*	*suff*		

Ly49G2,I,C

**Kb**	**RMS**	**Smin**	**Ly49G2**	**Ly49I**	**Ly49C**	**4**th strength	**4**th prob	**4**th expr
	5,07448	3	1	1	2	2	0,5	0,67
	5,07448	4	1	1	3	3	0,5	0,67
	5,07448	4	1	2	3	3	0,5	0,67
	5,07448	4	2	1	3	3	0,5	0,67
	5,07448	5	2	2	3	3	0,5	0,67
	5,07448	5	2	2	3	4	0,5	0,67
	5,07448	5	1	1	4	4	0,5	0,67
	5,07448	5	1	2	4	4	0,5	0,67
	5,07448	5	1	3	4	4	0,5	0,67
	5,07448	5	2	1	4	4	0,5	0,67
	5,07448	5	2	2	4	3	0,5	0,67
	5,07448	5	2	2	4	4	0,5	0,67
	5,07448	5	3	1	4	4	0,5	0,67
	5,07448	6	2	2	4	4	0,5	0,67
	5,07448	6	2	3	4	4	0,5	0,67
	5,07448	6	3	2	4	4	0,5	0,67
	5,07448	7	3	3	4	4	0,5	0,67
Kb			*insuff*	*insuff*	*insuff*	*insuff*		

**Db**	**RMS**	**Smin**	**Ly49G2**	**Ly49I**	**Ly49C**	**4**th strength	**4**th prob	**4**th expr
	5,64203	3	1	1	2	2	0,4	0,61
	5,64203	4	1	1	3	3	0,4	0,61
	5,64203	4	1	2	3	3	0,4	0,61
	5,64203	4	2	1	3	3	0,4	0,61
	5,64203	5	2	2	3	3	0,4	0,61
	5,64203	5	2	2	3	4	0,4	0,61
	5,64203	5	1	1	4	4	0,4	0,61
	5,64203	5	1	2	4	4	0,4	0,61
	5,64203	5	1	3	4	4	0,4	0,61

	5,64203	5	2	1	4	4	0,4	0,61
	5,64203	5	2	2	4	3	0,4	0,61
	5,64203	5	2	2	4	4	0,4	0,61
	5,64203	5	3	1	4	4	0,4	0,61
	5,64203	6	2	2	4	4	0,4	0,61
	5,64203	6	2	3	4	4	0,4	0,61
	5,64203	6	3	2	4	4	0,4	0,61
	5,64203	7	3	3	4	4	0,4	0,61
			insuff	*insuff*	*insuff*	*insuff*		

**Dd**	**RMS**	**Smin**	**Ly49G2**	**Ly49I**	**Ly49C**	**4th strength**	**4th prob**	**4th expr**
	5,35309	3	1	1	3	0,4		0,61
	5,35309	4	1	1	4	0,4		0,61
	5,35309	4	1	2	4	0,4		0,61
	5,35309	4	2	1	4	0,4		0,61
Dd			*insuff*	*insuff*	*insuff*	*suff*		

Ly49G2,I,C cont

**Ld**	**RMS**	**Smin**	**Ly49G2**	**Ly49I**	**Ly49C**	**4th strength**	**4th prob**	**4th expr**
	4,77798	2	1	1	0	1	0,4	0,55
	4,77798	3	1	2	0	2	0,4	0,55
	4,77798	3	2	1	0	2	0,4	0,55
	4,77798	3	2	2	0	1	0,4	0,55
	4,77798	3	2	2	0	2	0,4	0,55
	4,77798	4	1	3	0	3	0,4	0,55
	4,77798	4	2	2	0	2	0,4	0,55
	4,77798	4	2	2	0	3	0,4	0,55
	4,77798	4	2	3	0	2	0,4	0,55
	4,77798	4	2	3	0	3	0,4	0,55
	4,77798	4	3	1	0	3	0,4	0,55
	4,77798	4	3	2	0	2	0,4	0,55
	4,77798	4	3	2	0	3	0,4	0,55
	4,77798	4	3	3	0	1	0,4	0,55
	4,77798	4	3	3	0	2	0,4	0,55
	4,77798	4	3	3	0	3	0,4	0,55
	4,77798	4	2	2	1	2	0,4	0,55
	4,77798	5	1	4	0	4	0,4	0,55
	4,77798	5	2	3	0	3	0,4	0,55
	4,77798	5	2	3	0	4	0,4	0,55
	4,77798	5	2	4	0	3	0,4	0,55
	4,77798	5	2	4	0	4	0,4	0,55
	4,77798	5	3	2	0	3	0,4	0,55
	4,77798	5	3	2	0	4	0,4	0,55
	4,77798	5	3	3	0	2	0,4	0,55
	4,77798	5	3	3	0	3	0,4	0,55

	4,77798	5	3	3	0	4	0,4	0,55
	4,77798	5	3	4	0	2	0,4	0,55
	4,77798	5	3	4	0	3	0,4	0,55
	4,77798	5	3	4	0	4	0,4	0,55
	4,77798	5	4	1	0	4	0,4	0,55
	4,77798	5	4	2	0	3	0,4	0,55
	4,77798	5	4	2	0	4	0,4	0,55
	4,77798	5	4	3	0	2	0,4	0,55
	4,77798	5	4	3	0	3	0,4	0,55
	4,77798	5	4	3	0	4	0,4	0,55
	4,77798	5	4	4	0	1	0,4	0,55
	4,77798	5	4	4	0	2	0,4	0,55
	4,77798	5	4	4	0	3	0,4	0,55
	4,77798	5	4	4	0	4	0,4	0,55
	4,77798	5	2	3	1	3	0,4	0,55
	4,77798	5	3	2	1	3	0,4	0,55
	4,77798	5	3	3	1	2	0,4	0,55
	4,77798	5	3	3	1	3	0,4	0,55
	4,77798	6	2	4	0	4	0,4	0,55
	4,77798	6	3	3	0	3	0,4	0,55

Ly49G2,I,C cont

**Ld**	**RMS**	**Smin**	**Ly49G2**	**Ly49I**	**Ly49C**	**4th** strength	**4th** prob	**4th** expr
	4,77798	6	3	3	0	4	0,4	0,55
	4,77798	6	3	4	0	3	0,4	0,55
	4,77798	6	3	4	0	4	0,4	0,55
	4,77798	6	4	2	0	4	0,4	0,55
	4,77798	6	4	3	0	3	0,4	0,55
	4,77798	6	4	3	0	4	0,4	0,55
	4,77798	6	4	4	0	2	0,4	0,55
	4,77798	6	4	4	0	3	0,4	0,55
	4,77798	6	4	4	0	4	0,4	0,55
	4,77798	6	2	4	1	4	0,4	0,55
	4,77798	6	3	3	1	3	0,4	0,55
	4,77798	6	3	3	1	4	0,4	0,55
	4,77798	6	3	4	1	3	0,4	0,55
	4,77798	6	3	4	1	4	0,4	0,55
	4,77798	6	4	2	1	4	0,4	0,55
	4,77798	6	4	3	1	3	0,4	0,55
	4,77798	6	4	3	1	4	0,4	0,55
	4,77798	6	4	4	1	2	0,4	0,55
	4,77798	6	4	4	1	3	0,4	0,55
	4,77798	6	4	4	1	4	0,4	0,55
	4,77798	6	3	3	2	3	0,4	0,55
	4,77798	7	3	4	0	4	0,4	0,55
	4,77798	7	4	3	0	4	0,4	0,55

	4,77798	7	4	4	0	3	0,4	0,55
	4,77798	7	4	4	0	4	0,4	0,55
	4,77798	7	3	4	1	4	0,4	0,55
	4,77798	7	4	3	1	4	0,4	0,55
	4,77798	7	4	4	1	3	0,4	0,55
	4,77798	7	4	4	1	4	0,4	0,55
	4,77798	7	3	4	2	4	0,4	0,55
	4,77798	7	4	3	2	4	0,4	0,55
	4,77798	7	4	4	2	3	0,4	0,55
	4,77798	7	4	4	2	4	0,4	0,55
	4,77798	8	4	4	0	4	0,4	0,55
	4,77798	8	4	4	1	4	0,4	0,55
	4,77798	8	4	4	2	4	0,4	0,55
	4,77798	8	4	4	3	4	0,4	0,55
Ld			*insuff*	*insuff*	*no/insuff*	*insuff*		
